# A Comparison of Morphological and Molecular-Based Surveys to Estimate the Species Richness of *Chaetoceros* and *Thalassiosira* (Bacillariophyta), in the Bay of Fundy

**DOI:** 10.1371/journal.pone.0073521

**Published:** 2013-10-09

**Authors:** Sarah E. Hamsher, Murielle M. LeGresley, Jennifer L. Martin, Gary W. Saunders

**Affiliations:** 1 Biology, University of New Brunswick, Fredericton, New Brunswick, Canada; 2 Fisheries and Oceans Canada, St. Andrews Biological Station, St. Andrews, New Brunswick, Canada; George Washington University, United States of America

## Abstract

The goal of this study was to compare the ability of morphology and molecular-based surveys to estimate species richness for two species-rich diatom genera, *Chaetoceros* Ehrenb. and *Thalassiosira* Cleve, in the Bay of Fundy. Phytoplankton tows were collected from two sites at intervals over two years and subsampled for morphology-based surveys (2010, 2011), a culture-based DNA reference library (DRL; 2010), and a molecular-based survey (2011). The DRL and molecular-based survey utilized the 3′ end of the RUBISCO large subunit (*rbc*L-3P) to identify genetic species groups (based on 0.1% divergence in *rbc*L-3P), which were subsequently identified morphologically to allow comparisons to the morphology-based survey. Comparisons were compiled for the year (2011) by site (n = 2) and by season (n = 3). Of the 34 taxa included in the comparisons, 50% of taxa were common to both methods, 35% were unique to the molecular-based survey, and 12% were unique to the morphology-based survey, while the remaining 3% of taxa were unidentified genetic species groups. The morphology-based survey excelled at identifying rare taxa in individual tow subsamples, which were occasionally missed with the molecular approach used here, while the molecular methods (the DRL and molecular-based survey), uncovered nine cryptic species pairs and four previously overlooked species. The last mentioned were typically difficult to identify and were generically assigned to *Thalassiosira* spp. during the morphology-based survey. Therefore, for now we suggest a combined approach encompassing routine morphology-based surveys accompanied by periodic molecular-based surveys to monitor for cryptic and difficult to identify taxa. As sequencing technologies improve, molecular-based surveys should become routine, leading to a more accurate representation of species composition and richness in monitoring programs.

## Introduction

Planktonic marine diatoms are a key component to oceanic primary productivity, contributing ∼40% of the organic carbon generated in the sea each year [1], but their diversity is not well known [2], [3], [4]. As the stress on marine environments increases and species loss is more apparent, studies of phytoplankton diversity are becoming more important as a measure of ecosystem health [5].

Since 1987, the phytoplankton in the Bay of Fundy (BoF) have been monitored regularly by personnel at the Canadian Department of Fisheries and Oceans (DFO) to alert fisherman to harmful algal blooms (primarily *Alexandrium fundyense* Balech and species of *Pseudo-nitzschia* H. Perag. in H. & M. Perag.), establish a baseline of diversity, and determine seasonal trends and distribution of species [6], [7], [8], [9], [10], [11], [12]. These extensive monitoring efforts have allowed the DFO to track the introduction of new species into the flora and density changes in harmful algal bloom-forming species [10]. Two of the most species-rich and abundant diatom genera in the BoF are *Chaetoceros* spp. and *Thalassiosira* spp. [6], [7], [8], [9], [11], [12]. These genera are widespread, difficult to identify due to the morphological similarity of their constituent species, and each genus is known to contain >100 species [13], [14], [15]. However, their identification in the BoF has been based solely on morphology.

Two molecular surveys of planktonic diatoms in the BoF have been published previously [3], [16]. Savin et al. compared morphological and molecular methods of measuring plankton diversity in the BoF and found little overlap (5%) between methodologies: while diatoms were the taxa most frequently encountered in the morphological surveys, only three of the 64 sequences generated were attributed to them [3]. In fact, many of the sequences in that study were not closely related to known organisms, making comparisons between the two methodologies difficult [3]. However, their method of using universal primers to amplify DNA from an environmental sample would presumably lead to PCR bias and large numbers of sequences from smaller organisms that would be missed by the morphological survey. Kaczmarska et al. conducted a molecular survey of *Pseudo-nitzschia delicatissima* (Cleve) Heiden, a diatom that produces domoic acid in some regions in eastern Canada, including four sites in the BoF, and concluded that their isolates belonged to a single metapopulation lacking phylogeographic pattern [16].

In contrast to the results for *Pseudo-nitzschia delicatissima* by Kaczmarska et al., molecular surveys for other diatom species (e.g., *Navicula cryptocephala* Kütz., *Nitzschia palea* (Kütz.) W. Sm., *Pseudo-nitzschia* spp., *Sellaphora pupula* (Kütz.) Mereschk., etc.) in other geographic areas have revealed ‘cryptic’ species that are reproductively and genetically distinct, but with virtually identical morphology ([16], [17], [18], [19], [20], respectively) making accurate identification without molecular data difficult to impossible for these species complexes [21]. These cases of ‘cryptic’ diversity can confound monitoring efforts leading to an underestimation of species richness and could foreseeably result in overlooking introduction events for taxa from such cryptic complexes.

The application of contemporary molecular tools to routine monitoring efforts can effectively resolve the issues presented by cryptic complexes leading to more accurate estimates of species richness and plankton community composition. The most common molecular markers used to identify diatoms are based on genes from genomes of three cellular compartments including: the 5′ end of the cytochrome *c* oxidase I gene (COI-5P) as a mitochondrial marker; the RUBISCO large subunit (*rbc*L) and the universal plastid amplicon (UPA) as plastid markers; and the large subunit ribosomal DNA (LSU), the small subunit ribosomal DNA (SSU), and the internal transcribed spacer (ITS) as nuclear markers [18], [20], [22], [23], [24], [25], [26]. While the shorter, less variable markers, such as UPA and SSU, have greater utility for identifying a diversity of protistan taxa in broad surveys [27], [28], they are too conserved to be able to distinguish between closely related diatom species [23], [26]. The highly variable COI-5P and ITS regions are able to distinguish between diatom species, but either cannot be easily amplified and sequenced for some taxa (COI-5P, [25]) or can display intraclonal variability that makes reading the sequences of some species difficult to impossible without cloning (ITS, [29]). Owing to the previous issues, Hamsher et al. advocated the use of a 780-bp region near the 3′ end of the *rbc*L (*rbc*L-3P) as well as the variable D2/D3 region of the LSU (LSU D2/D3) as species-level markers for diatom identification [25]. Each marker can be easily amplified and sequenced for many genera using a single marker-specific primer pair and in combination they provide adequate resolving power to differentiate between closely related diatom species [25].

The primary objective of this study was to compare the ability of morphological and molecular techniques (*rbc*L-3P data) to identify *Chaetoceros* and *Thalassiosira* during monitoring surveys and assess the strengths and limitations of each approach. To accomplish these goals, species of *Chaetoceros* and *Thalassiosira* from the BoF were isolated into unialgal culture to develop a DNA reference library (DRL). Cultures in the DRL were sequenced (*rbc*L-3P and LSU D2/D3 data) and morphologically identified so that sequences generated during the subsequent molecular-based survey could be tied to known species. For the molecular-based survey, single colonies were isolated, sequenced, and overall species richness recorded. These data were then compared to the species composition and richness recorded from the routine morphology-based survey for the same samples.

## Methods

### Ethics Statement

No permits were required for collection of water samples in Canadian coastal waters.

### Sample Collection

Plankton tows were collected at Passamaquoddy Bay (PB; 45.067N, −66.967W) and, starting in April 2010, the Wolves (WV; 45.000N, −66.733W), in the BoF in 2010–2011. In 2010, tows were collected monthly during the winter months (Jan–Apr, Oct–Dec) and weekly during the summer months (May–Sept) resulting in 59 tows. In 2011, tows were collected at a similar interval (monthly in January and April; twice in May; weekly from June-September; twice in October; and monthly in November and December) resulting in 46 tows. All tows were collected from the CCGS *Pandalus III* or the *Viola M. Davidson*. At each site, a 10 m vertical plankton haul was collected with a 20-µm mesh plankton net, 0.3 m in diameter. Organisms were flushed into a collection container. Plankton tows were kept in the dark on ice until returning to the lab. All tows were subsampled for routine monitoring purposes (see *Morphology-based survey* below) and either development of a culture-based DRL for 2010 tows or for the molecular-based survey for 2011 tows. For the DRL, tow subsamples were immediately brought back to UNB on ice. For the molecular-based survey, each tow subsample was settled in a 50 mL Falcon**™** tube for 2–4 hours, the supernatant was then removed by reverse osmosis filtration, and finally 95% ethanol was added for cell preservation. These concentrated samples were stored at −20°C until the molecular-based survey was completed.

### Morphology-based Survey

Approximately two drops of each tow subsample was examined at 200–400X using a Nikon Eclipse 50*i* light microscope with phase contrast. Phytoplankton were identified live within four hours of sampling and their abundance noted using the Helsinki Commission (HELCOM, [30]) rating scale (i.e., 1 =  very sparse/rare; 2 =  sparse; 3 =  scattered/common; 4 =  abundant; 5 =  dominant). Species discovery curves were used to determine when examination of a tow subsample should discontinue. Diatoms were identified using the following references: Bérard-Therriault et al., Horner, Hoppenrath et al., Jensen & Moestrup, Kraberg et al., Rines & Hargraves, Throndsen et al., and Tomas [31], [32], [33], [34], [35], [36], [37], [38]. Due to the semi-quantitative abundance data, results discussing abundance were based on the number of plankton tow subsamples in which a particular species occurred (n = 46 in 2011) and the number of tow subsamples for which a species had a HELCOM rating of >1. Only data for the colony-forming species of *Chaetoceros* and *Thalassiosira* were included in this study. Due to the low magnification (320X) used for isolating colonies during development of the DRL and the molecular-based survey (see below), the following species of these genera were excluded from comparisons between morphology and molecular-based surveys because they do not form colonies with >2 cells (i.e., easily visible colonies at 320X) in the BoF: *C. convolutus* f. *trisetosa* Brunel, *C. danicus* Cleve, and *C. simplex* Ostenf. One additional taxon, *C. socialis* Lauder, was excluded because, while it was common in the flora and in the DRL, colony structure degenerated during ethanol preservation making inclusion of this taxon in the molecular-based survey (below) not feasible with the techniques employed in this study.

### Development of the Culture-based DNA Reference Library (DRL)

**Figure 1:**
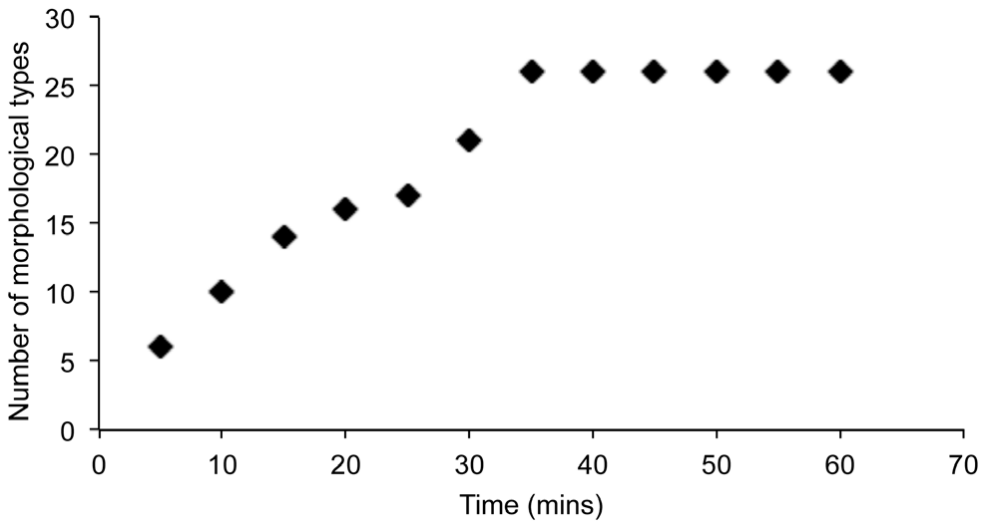
Example of a morphological species-discovery curve used to determine when colony isolation should discontinue. This curve was generated for colonies isolated from the Passamaquoddy Bay plankton tow subsamples on June 1, 2010 for development of the culture-based DNA reference library (DRL). Isolation was terminated after 60 mins (n = 42 colonies of 26 morphological species isolated) because 30 mins had elapsed with no new morphological types found.

Colonies were isolated from each of the tow subsamples collected in 2010 as follows. In the laboratory, ∼200 µL of the tow subsample was diluted with sterile seawater in a Petri dish. Individual phytoplankton colonies were rudimentarily identified (i.e., identified to ‘species group’ such as *Chaetoceros diadema/decipiens* or large *Thalassiosira*) using a dissecting scope at 320X and subsequently isolated into F/2 liquid media using a glass micropipette. Species-discovery curves (see example curve, Fig. 1) were generated for each tow subsample to determine when identification and isolation of cells should discontinue. All cultures were maintained at 10°C in growth chambers with 8∶16 for January-April and October 19-Dec isolates or 12∶12 for May-October 12 isolates L:D. Morphological species that were not successfully entered into culture from one tow subsample were sought after during the next sampling period. Cultures were partially harvested and preserved within a month of isolation for morphological identification with formalin-acetic acid (FAA; 420 µL concentrated culture: 600 µL of 70% ethanol: 120 µL commercial formalin: 60 µL glacial acetic acid [39]). Preserved cultures have been deposited in the Connell Memorial Herbarium (UNB) and representative cultures of most genetic species groups have been deposited in the Culture Collection of Algae and Protozoa (CCAP) (see Table S1 for details).

A subsample of each culture (Table S1) was harvested by centrifugation for DNA extraction. The DNA extraction followed the diatom protocol outlined in Saunders & McDevit with modification to account for low sample volume (samples were extracted in 200 µL of brown algal buffer and DNA was eluted in 100 µL of TE) or the Chelex protocol described by Richlen & Barber [40], [41]. Extracted DNA from a majority of species has been deposited at the Ocean Genome Legacy (SE23396–SE23435, [42]). Data for the *rbc*L-3P and LSU D2/D3 regions were generated following Hamsher et al. (2011) [25]. Sequences were assembled and edited using Sequencher™ 4.8 (Gene Codes Corporation, Ann Arbor, MI, USA) and aligned by eye in MacClade 4 (Version 4.06) for OSX [43]. The final alignment of *rbc*L-3P sequences included data from 184 cultures (780 bp with no indels). The LSU D2/D3 alignment had data from 173 cultures (834 sites). Cluster analyses were preformed on each alignment separately using the neighbor-joining algorithm on uncorrected dissimilarities (p) in Geneious v5.4 [44] to identify genetic species groups for the DRL.

Following genetic species assignment, preserved material from representatives of each genetic species group was examined at 1000X using a Leica DM5000B light microscope and photographed using a Leica DFC480 digital camera (Leica, Heidelberg, Germany). In some cases (e.g., some *Thalassiosira* species), morphological features were obscured by the presence of the cell contents so preserved material was boiled in H_2_O_2_ for 30 mins, systematically settled and washed with ddH_2_O, dried to coverslips, and mounted with Naphrax® for identification. References utilized to identify these diatoms were similar to those used during the morphology-based survey (i.e., [32], [33], [35], [36], [38]).

### Molecular-based Survey

The 2011 preserved tow subsamples (50 mL) were subsampled again (300 µL) and these were combined for each season based on the abundance of phytoplankton: winter included January, April, May (winter); summer included June through September (summer); and fall included October through December (fall). From six samples in total (i.e., one from each of two sites for each of three seasons), individual colonies of *Chaetoceros* spp. and *Thalassiosira* spp. were isolated into microtubes for DNA extraction. Individual colonies were isolated following the species-discovery curve methodology described above. A sketch/description of each isolated colony was recorded to facilitate the subsequent identification in case the sequence generated did not match data in the DRL. After the ethanol was allowed to evaporate, 20 µL of a 10% (w/v) Chelex solution was added to each isolated colony and the DNA extracted following Richlen & Barber [41]. The *rbc*L-3P region was amplified and sequenced in the forward direction following Hamsher et al. [25]. Resulting sequences were compared to the DRL and their affinity noted. Colonies lacking a match to the DRL were identified based on the notes taken during isolation. A representative sequence from each genetic species group (from both the DRL and novel groups uncovered during the molecular-based survey) was included in an alignment as described above (46 sequences with 780 bp, no indels) and cluster analysis performed as above. Resulting sequences from this study (from cultures and single colony isolations) have been deposited in BOLD (www.boldsystems.org) and GenBank (see Table S1 for BOLD identifiers and accession numbers, respectively).

## Results

### Morphology-based Survey

During 2010, 20 *Chaetoceros* spp. and seven *Thalassiosira* spp. were identified. Of the 20 *Chaetoceros* spp., 20% were unique to a single tow subsample. *Chaetoceros debilis* Cleve was the most abundant *Chaetoceros*: this taxon was present in the greatest number of tow subsamples (n = 29) and had the greatest number of tow subsamples with a HELCOM rating >1 (n = 15; Table 1). In contrast, all of the *Thalassiosira* spp. identified occurred in multiple tow subsamples with only two species, *T. bioculata* var. *bioculata* (Grunow) Ostenf. and *Thalassiosira* tiny sp., in fewer than five tow subsamples (Table 1). The most abundant was *Thalassiosira gravida* Cleve (n = 37 tow subsamples and n = 8 with HELCOM rating >1; Table 1). Only 16% (4) of taxa were unique to Passamaquoddy Bay or the Wolves and these were only found in single tow subsamples at low abundance. *Thalassiosira* spp. were present throughout 2010, while most *Chaetoceros* spp. were present only from May to October 2010.

**Table 1:** *Chaetoceros* and *Thalassiosira* diversity and abundance as recorded during the morphology-based surveys in 2010 and 2011.

Year/Taxon	n^a^		Months^b^		n>1^c^		months n>1^d^	
	WV	PB	WV	PB	WV	PB	WV	PB
2010								
*Chaetoceros affinis*	1	2	Jul	Jul	0	0	–	–
*Chaetoceros atlanticus*	1	0	A	–	0	0	–	–
*Chaetoceros borealis*	1	0	Jul	–	0	0	–	–
*Chaetoceros concavicornis*	0	1	–	M	0	0	–	–
*Chaetoceros constrictus*	1	4	Jun	Jun, Aug	0	1	–	Jun
*Chaetoceros contortus*	12	9	Jun-S	M, Jun-S	4	5	Jun, S	Jun, Jul
*Chaetoceros convolutus*	4	8	A, Jun, S	M, Jun, S, O	1	1	S	S
*Chaetoceros debilis*	13	16	A-Jun, S-N	J, M, A- Jun, S, O	7	8	My, Jun, S	M, My, Jun, S
*Chaetoceros decipiens*	7	4	My, Jun, Aug, S, O	M, My, Jun, S	0	0	–	–
*Chaetoceros diadema*	3	3	My, Jun	My, Jun	1	0	Jun	–
*Chaetoceros didymus*	5	5	My, Jul, S, O	Jul-S	1	2	Jul	Jul
*Chaetoceros ingolfianus*	1	2	My	My, Jun	1	1	My	My
*Chaetoceros furcellatus*	2	2	My	My, Jun	2	1	My	My
*Chaetoceros laciniosus*	7	3	My–Aug	My, Jul, Aug	1	0	My	–
*Chaetoceros lorenzianus*	4	8	Jul, S	Jul-O	0	0	–	–
*Chaetoceros radicans*	2	2	Jun, Jul	Jun, Jul	0	1	–	Jun
*Chaetoceros similis*	2	9	Aug, S	J-M, Jun, Aug-O	0	1	–	M
*Chaetoceros simplex*	0	1	–	Jun	0	0	–	–
*Chaetoceros socialis*	13	12	A-Jul, S	A-O	5	8	My–Jul	My–Jul
*Chaetoceros teres*	4	4	A, My	M, My	0	1	–	My
*Thalassiosira auguste-lineata*	13	5	A-Aug, N	A-Jun, N	2	2	Jun	Jun
*Thalassiosira baltica*	4	2	Jun, Jul, N	N, D	2	0	Jun, Jul	–
*Thalassiosira bioculata var. bioculata*	1	2	Jun	Jun	0	0	–	–
*Thalassiosira gravida*	16	21	My-S	J-M, My-O	3	5	Aug, S	Jun-S
*Thalassiosira nordenskioeldii*	7	10	A-Jun	F-Jun	4	3	A, My	A-Jun
*Thalassiosira punctigera*	17	15	Jun-D	J-M, Jun, Jul, S-D	4	1	Jun, Jul, O	O
*Thalassiosira* tiny sp.	3	1	Jul, S, N	F	1	0	Jul	–
2011								
*Chaetoceros affinis*	0	2	–	Aug	0	0	–	–
*Chaetoceros concavicornis*	1	0	N	–	0	0	–	–
*Chaetoceros contortus*	4	4	Jun–Aug	Jun–Aug	1	1	Jun	Aug
*Chaetoceros convolutus*	2	1	A	A	0	0	–	–
*Chaetoceros debilis*	12	14	Jun, Aug, S, O	A-O, D	4	2	Jun, Aug, S	Jun, O
*Chaetoceros decipiens*	9	13	J, A, Jun-O	A-S, N	0	2	–	Jun
*Chaetoceros diadema*	0	2	–	Jun	0	0	–	–
*Chaetoceros didymus*	4	7	Aug, O	Jun-O	0	3	–	Jul, Aug
*Chaetoceros furcellatus*	1	1	Jun	My	0	0	–	–
*Chaetoceros laciniosus*	5	5	A, Jun, Jul	My, Jun, Aug, N	0	0	–	–
*Chaetoceros lorenzianus*	1	5	Aug	Jul-O	0	0	–	–
*Chaetoceros pseudobrevis*	1	1	Aug	Aug	0	0	–	–
*Chaetoceros radicans*	4	3	Jun–Aug	Jun, Aug	3	0	Jul, Aug	–
*Chaetoceros similis*	6	12	Jun, S, O	A- Jun, Aug-D	0	0	–	–
*Chaetoceros socialis*	5	11	Jun–Aug, O	Jun-S	3	6	Jun, Jul	Jul-S
*Thalassiosira auguste-lineata*	11	10	A, Jun, Aug-D	J, A, Jun–Aug, N, D	0	0	–	–
*Thalassiosira baltica*	3	2	Jun, D	A, N	0	0	–	–
*Thalassiosira bioculata* var. *exigua*	1	0	N	–	0	0	–	–
*Thalassiosira gravida*	17	14	A, Jun-D	A, Jun-D	5	5	Jul, Aug, O	Jul, Aug
*Thalassiosira nordenskioeldii*	3	4	A, Jun	A, Jun	1	0	A	–
*Thalassiosira punctigera*	12	7	J, Jun, Aug-D	J, My, S-D	2	3	O, D	O, N, D
*Thalassiosira* sp. (tiny)	4	1	Jun, Aug, N, D	Jun	0	0	–	–

PB = Passamaquoddy Bay; WV = The Wolves; J = January; F = February; M = March; A = April; My = May; Jun = June; Jul = July; Aug = August; S = September; O = October; N = November; and D = December.

a n indicates the number of tow subsamples in which each taxon was present (n = 59 for 2010 and n = 46 for 2011).

b Months are the months each taxon was present.

c n>1 are the number of tow subsamples with a HELCOM rating>1.

d Months n>1 are the months in which the higher abundance occurred.

In 2011, fifteen *Chaetoceros* and seven *Thalassiosira* spp. were identified. Only four species, *Chaetoceros affinis* Lauder, *C. concavicornis* L. Mangin, *C. diadema* (Ehrenb.) Gran., and *Thalassiosira bioculata* var. *exigua* (Grunow) Hust., were restricted to one site and in low abundance when present (i.e., fewer than three tow subsamples and none with HELCOM ratings>1; Table 1). Again, *C. debilis* (n = 26 tow subsamples, n = 6 with HELCOM rating>1) and *T. gravida* (n = 31 tow subsamples, n = 10 with HELCOM rating>1; Table 1) were the most abundant taxa. A majority (69%) of taxa reported in 2010 were also found in 2011 (Table 2). Almost all of the taxa unique to 2010 (n = 7) or 2011 (n = 2) sampling years were *Chaetoceros* spp. and five of these were at low abundance (i.e., present in fewer than four tow subsamples, none with HELCOM ratings>1; Table 1).

**Table 2:** *Chaetoceros* and *Thalassiosira* species reported in the Bay of Fundy.

	Monitoring Years										
Taxon	86–87	88–89	90–92	93–96	97–98	99–00	2010	2011	2010DRL^a^	2011MolecularSurvey^b^	TotalGeneticspecies^c^
*Chaetoceros affinis*	–	X	X	X	X	–	X	X	–	–	–
*Chaetoceros atlanticus*	X	–	X	X	X	–	X	–	–	–	–
*Chaetoceros borealis*	X	–	X	X	X	X	X	–	–	–	–
*Chaetoceros concavicornis*	–	–	–	–	X	X	X	X	–	1	1
*Chaetoceros constrictus*	X	X	X	X	X	X	X	–	1	1	1
*Chaetoceros contortus* d	X	X	X	X	X	X	X	X	–	2	2
*Chaetoceros convolutus*	X	X	X	X	X	X	X	X	–	–	–
*Chaetoceros debilis*	X	X	X	X	X	X	X	X	2	2	2
*Chaetoceros decipiens*	X	X	X	X	X	X	X	X	1	2	2
***Chaetoceros densus***	–	–	–	X	–	–	–	–	–	–	–
*Chaetoceros diadema*	X		X	X	X	X	X	X	2	2	2
*Chaetoceros didymus*	X	X	X	X	X	X	X	X	1	1	1
***Chaetoceros diversus***	–	X	–	–	–	–	–	–	–	–	–
*Chaetoceros furcellatus*	X	X	X	X	X	X	X	X	–	–	–
*Chaetoceros ingolfianus*	–	–	–	X	X	X	X	–	–	–	–
***Chaetoceros karianus***	X	–	–	–	–	–	–	–	–	–	–
*Chaetoceros laciniosus*	X	X	X	X	X	X	X	X	1	2	2
*Chaetoceros lorenzianus*	X	X	X	X	X	X	X	X	–	2	2
*Chaetoceros mitra*	X	–	X	–	–	–	–	–	–	–	–
*Chaetoceros perpusillus*	X	–	–	–	X	–	–	–	–	–	–
*Chaetoceros pseudobrevis* e	–	–	–	X	X	–	–	X	2	1	2
*Chaetoceros radicans*	–	X	–	–	–	X	X	X	1	1	2
*Chaetoceros similis*	–	–	–	–	X	X	X	X	1	–	1
*Chaetoceros simplex*	–	X	X	X	X	X	X	–	1	–	1
*Chaetoceros socialis*	–	X	X	X	X	X	X	X	1	–	1
*Chaetoceros subtilis*	–	X	–	X	X	X	–	–	–	–	–
*Chaetoceros teres*	X	X	X	X	X	X	X	–	1	–	1
*Chaetoceros willei*	X	X	–	–	–	–	–	–	–	–	–
*Thalassiosira angulata*	–	–	–	X	X	–	–	–	1	1	1
*Thalassiosira antarctica*	–	–	–	–	–	–	–	–	1	1	1
*Thalassiosira auguste-lineata*	–	–	–	X	X	X	X	X	1	1	1
*Thalassiosira baltica*	–	X	X	X	X	X	X	X	–	1	1
*Thalassiosira bioculata* var. *bioculata*	–	–	–	–	–	–	X	–	–	–	–
*Thalassiosira bioculata* var. *exigua*	–	–	–	–	–	–	–	X	–	1	1
*Thalassiosira condensata*	X	X	X	–	–	–	–	–	–	–	–
*Thalassiosira decipiens*	X	X	X	–	X	X	–	–	1	–	1
*Thalassiosira delicata*	–	–	–	–	–	–	–	–	1	1	1
*Thalassiosira eccentrica*	–	–	–	–	–	–	–	–	2	1	2
*Thalassiosira gravida*	X	X	X	X	X	X	X	X	1	1	1
*Thalassiosira nordenskioeldii*	X	X	X	X	X	X	X	X	1	1	1
*Thalassiosira oestrupii*	–	–	–	–	X	X	–	–	–	–	–
*Thalassiosira pacifica*	–	–	–	–	–	–	–	–	1	1	1
*Thalassiosira punctigera*	–	–	–	–	–	–	X	X	1	1	1
*Thalassiosira* tiny sp.	–	–	–	–	–	–	X	X	–	1	1
*Thalassiosira subtilis*	X	X	X	–	–	–	–	–	–	–	–
Unknown *Thalassiosira*	–	–	–	–	–	–	–	–	–	1	1
TOTAL number of species	22	23	23	25	29	25	27	22	26	30	38

*Chaetoceros* and *Thalassiosira* reports based on Wildish et al. (1988, 1990), Martin et al. (1995, 1999, 2001, 2006) and the current 2010–2011 morphology-based surveys, the 2010 DRL, and the 2011 molecular-based survey. Bold highlights taxa recorded in only one year. DRL = DNA reference library.

X = presence; – = absence.

a Number of genetic species groups assignable to each morphology-based species based on the 2010 DRL.

b Number of genetic species groups assignable to each morphology-based species uncovered during the 2011 molecular-based survey.

c Total number of genetic species uncovered during this study for each morphological species.

d This taxon was recorded as *Chaetoceros compressus* in the 86–00 monitoring years.

e This taxon was recorded as *Chaetoceros brevis* in the 93–96 and 97–98 monitoring years.

### Culture-based DNA Reference Library (DRL)

**Figure 2:**
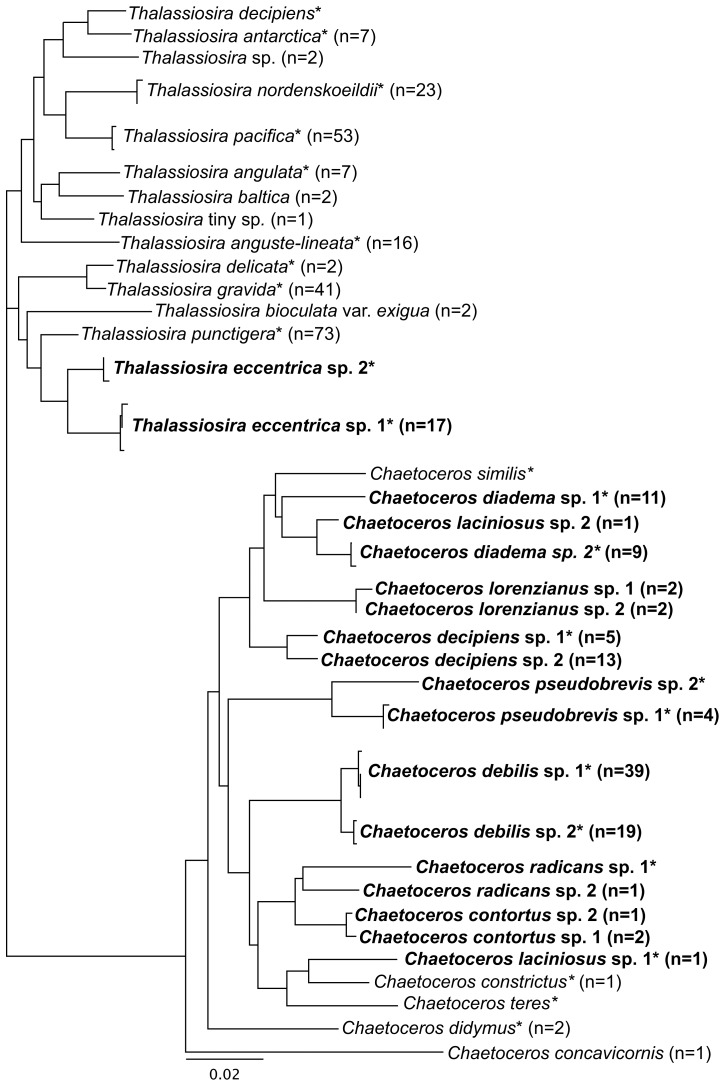
Diversity of genetic species groups displayed as an unrooted phylogram inferred from the *rbc*L-3P data. An asterisk indicates species included in the 2010 culture-based DNA reference library (DRL). n indicates the number of sequences generated for each genetic species group during the 2011 molecular-based survey. If no number is indicated then this taxon was encountered in the 2010 DRL only. Cryptic species pairs are indicated in bold font.

The DRL served as a bridge between the morphological and molecular assessments by tying sequence data to known taxa. Cultures were analyzed primarily using *rbc*L-3P data (Fig. 2), with LSU D2/D3 data (Table S1) generated for at least one representative of most *rbc*L-3P genetic species groups to confirm the *rbc*L-3P results. The within genetic species divergence threshold was set at 0.1% (0–1 bp) for *rbc*L-3P based on Trobajo et al. [45]. At this level of divergence, *rbc*L-3P and LSU D2/D3 data agreed on delineation of genetic species groups. While this level of divergence is slightly higher than that reported between closely related *Sellaphora* spp. (0.1%, [25]), making an underestimate of diversity possible, it will not change the overall conclusions of our work as no taxonomic conclusions are drawn, and it is better to under-estimate than over-estimate diversity for the objectives of this study.

Based on this threshold, 51 *Chaetoceros* and 141 *Thalassiosira* cultures successfully isolated and sequenced for inclusion in the DRL resolved into 13 and 11 genetic species groups, respectively (Fig. 2, asterisk) from the 2010 tow subsamples (n = 59). Of the 27 species of *Chaetoceros* and *Thalassiosira* identified during the morphology-based survey in 2010, 56% (n = 15) were represented in the DRL. Of the 11 species not represented in culture, only six had a HELCOM rating>1 in any single tow subsample (*C. contortus* F. Schütt, *C. convolutus* Castracane, *C. furcellatus* J.W. Bailey, *C. ingolfianus* Ostenf. *Thalassiosira* tiny sp., and *T. baltica* (Grunow) Ostenf.). The remaining species (i.e., *C. affinis* Lauder, *C. atlanticus* Cleve, *C. borealis* J.W. Bailey, *C. concavicornis*, *C. lorenzianus* Grunow and *T. bioculata*) never had a HELCOM rating>1 and almost all of these species, except *C. lorenzianus* and *T. bioculata*, were reported only in a single tow subsample in 2010. Seven species were identified from the DRL that were not recorded during the morphology-based survey. Three of these unreported taxa (i.e., *C. pseudobrevis* Pavill., *T. angulata* (W. Greg.) Hasle, and *T. decipiens* (Grunow) E.G. Jørg.) were recorded in previous years, but were not in 2010 (Table 2). Some of the unreported taxa may have been rare in the field because they were represented by only a few cultures (<5; Table S1) including: *C. pseudobrevis*, *T. angulata*, *T. antarctica* Comber, *T. decipiens*, and *T. delicata* (J.A. Barron) Akiba. However, two taxa were common in the DRL and possibly misidentified in the morphology-based survey, including: *T. eccentrica* (Ehrenb.) Cleve (n = 27 cultures), which may have been mistaken for *T. nordenskioeldii* Cleve; and *T. pacifica* Gran & Angst (n = 29 cultures), which may have been identified as *T. punctigera* (Castracane) Hasle (Table S1). The culturing efforts also uncovered four cases of possible cryptic diversity: each morphological taxon had two genetic species groups based on *rbc*L-3P and LSU D2/D3 data. The four cryptic species pairs were for the morpho-species *C. debilis*, *C. diadema*, *C. pseudobrevis*, and *T. eccentrica* and members of each species pair were often represented by more than one culture (Fig. 2, see Table S1). In contrast, no single genetic species group was found to include more than one morphospecies based on the representatives examined.

### Molecular-based Survey

From the six combined tow subsamples for 2011 (three seasons at two sites), 17 *Chaetoceros* and 13 *Thalassiosira* genetic species groups were identified based on *rbc*L-3P data (Fig. 2) including eight *Chaetoceros* and four *Thalassiosira* genetic species groups that were not present in the DRL. The identification of these 12 additional taxa was based on the sketches/notes taken during colony isolation. Ten of these genetic species groups could be identified to the species level (or variety) and included: *C. concavicornis*, *Chaetoceros contortus* Lauder (sp. 1 and 2), *C. decipiens* sp. 2, *C. laciniosus* Schütt sp. 2, *C. lorenzianus* (sp. 1 and 2), *C. radicans* F. Schütt sp. 2, *T. baltica*, and *T. bioculata* var. *exigua* (Fig. 2). Of the two remaining groups, one (*Thalassiosira* tiny sp.) matched the description of a morphological species in the morphology-based survey and the other could not be identified to species based on the sketch/notes taken (recorded simply as *Thalassiosira* sp.) (Fig. 2).

A few interesting trends were uncovered when comparing the cryptic species in the 2010 DRL to those identified in the 2011 molecular-based survey. For example, for some cryptic species pairs one partner of the pair was present in the 2010 DRL, but not in the 2011 molecular-based survey and vice versa (e.g. *Chaetoceros radicans*; Table S1). Other cryptic species pairs were both present in the 2010 DRL, but with only of the pair found in the 2011 molecular-based survey (e.g., *C. pseudobrevis*; Table S1) and vice versa (e.g., *C. laciniosus*; Table S1). Based on the molecular-based survey data, a few of these cryptic taxa may be seasonal, which has substantial consequences for the morphology-based surveys. For example, at site PB *Chaetoceros debilis* sp. 1 was not found in summer, but both *C. debilis* sp. 1 and 2 were found in fall; *Chaetoceros decipiens* sp. 2 was found throughout the year, but *C. decipiens* sp. 1 was only found in fall; and *Chaetoceros diadema* sp. 2 was found in winter and summer while *Chaetoceros diadema* sp. 1 was found only in fall. The isolation date of the cultures in the DRL (2010) support these trends, except for two cases: *C. decipiens* sp. 1 and *C. diadema* sp. 1 were isolated in September instead of only fall, perhaps due to differences in temperature between years. All of the previous observations would go unnoticed for these cryptic species pairs in the morphology-based survey.

### Comparison of Morphology and Molecular-based Surveys to Estimate Species Richness

**Figure 3:**
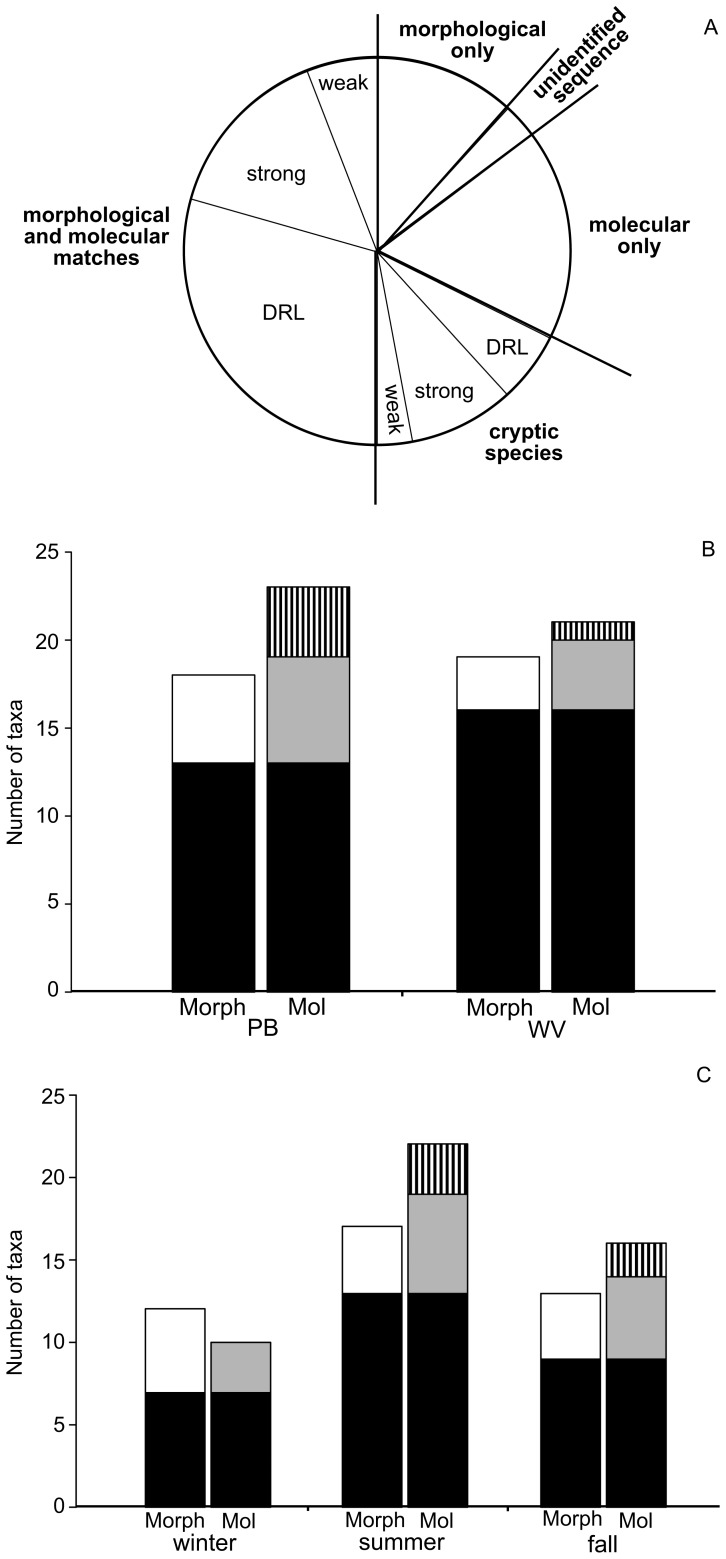
Comparisons between the number of species found during the morphology (morph) and molecular-based (mol) surveys. Data were summarized for the 2011-monitoring year (**A**), and then by site (**B**), and by season (**C**). In the year summary (**A**), taxa were placed into five main categories in bold font (two of these were divided further into three subcategories each) as indicated on the figure and described in the text. In the by site (**B**) and by season (**C**) summaries, the taxa are placed in only three categories including taxa that were identified: in common to the morphology (morph) and molecular (mol) -based surveys (black); or in the morphology-based survey only (white); in the molecular-based survey only as unique (gray) or cryptic (vertical lines) species. Sites were Passamaquoddy Bay (PB) and The Wolves (WV). Seasons were Jan, Apr, May (winter), June–Sept (summer), and Oct–Dec (fall).

A comparison of the number of taxa identified during the morphology-based versus the molecular-based surveys was compiled for 2011 (Fig. 3). Taxa were placed into one of five categories. The largest category, 50% of taxa, included taxa that were identified in both the morphology-based and molecular-based surveys (Fig. 3A). This category was separated further into subcategories based on the confidence we could place in the match of the molecular to the morphological species. The most confident matches, and also the largest subcategory (29% of all taxa), included taxa that were in the DRL (Fig. 3A). All of these taxa were recorded in the 2011 morphology-based survey in more than one tow subsample (Table 1). The next subcategory (15% of all taxa) included those taxa whose match could be confidently surmised from the sketches drawn during isolation of individual colonies during the 2011 molecular-based survey (Fig. 3A, strong). The subcategory with the lowest confidence (6% of all taxa) included taxa whose match was also predicted based on the sketch drawn during isolation (*T. bioculata* var. *exigua* and *Thalassiosira* tiny sp.), but their match was less certain than those in the previous category (Fig. 3A, weak).

The second category (3% of all taxa) was designated for a single taxon, *Thalassiosira* sp., whose basic morphology and resulting sketch were too general to tie this genetic species to a morphological taxon and so was recorded as an unidentified sequence (Fig. 3A).

The third category included taxa that were identified only during the morphology-based survey (12% of all taxa, Fig. 3A, morphological only) and was limited to four species of *Chaetoceros* (i.e., *C. affinis, C. convolutus, C. furcellatus* and *C. similis* Cleve) that were reported in fewer than five tow subsamples (except for *C. similis* found in 18 tow subsamples) and never in high abundance (i.e., no tow subsamples with HELCOM ratings>1, including *C. similis*; Table 1).

The fourth category included taxa unique to the molecular-based survey that were nonetheless included in the DRL and thus were assigned a morphological species designation (17% of all taxa, Fig. 3A, molecular only). Most of the species missed during the morphology-based survey (Fig. 3A, molecular only) were *Thalassiosira* spp. (*T. angulata, T. antarctica, T. decipiens, T. eccentrica,* and *T. pacifica*), as well as *Chaetoceros constrictus* Gran.

The final category was for cryptic species and included 18% of all taxa (Fig. 3A). Cryptic species uncovered always occurred in pairs and thus one partner of each pair was considered a match (Fig. 3A, morphological and molecular match) and the other a cryptic species in the summary presentation (Fig. 3A). Each cryptic partner (i.e., the partner of the cryptic species pair not considered a morphological and molecular match) was again placed into one of three subcategories based on the confidence of their morphological identification: 1) the cryptic partner was uncovered previously during the development of the DRL and thus the morphological comparison was detailed in support of the cryptic designation (6% of all taxa, Fig. 3A, DRL); 2) the sketch of the cryptic partner drawn during isolation for the molecular-based survey was sufficiently detailed to allow for a strong morphological association (9% of all taxa, Fig. 3A, strong); and 3) a single cryptic partner, *C. laciniosus* sp. 2, that is likely a cryptic species, but whose sketches were insufficient to render a solid morphological identification (3% of all taxa, Fig. 3A, weak). All of the cryptic species pairs found during these comparisons were attributed to *Chaetoceros* spp. (*C. contortus* (n = 2), *C. debilis* (n = 2), *C. decipiens* (n = 2), *C. diadema* (n = 2), *C. laciniosus* (n = 2) and *C. lorenzianus* (n = 2)).

To compare differences between sites (PB and WV) and seasons (winter, summer, fall) between the morphological and molecular-based surveys the data were lumped according to the main categories in Figure 3A: morphological and molecular matches (Figs 3B and 3C, black): cryptic species (Figs 3B and 3C, vertical lines): morphological based-survey only (Figs 3B and 3C, white): and molecular-based survey only (Figs 3B and 3C, grey) (Fig. 3A, unidentified sequence was removed from further comparisons). Cryptic taxa were only recorded as such in the ‘by site’ and ‘by season’ comparisons if both partners (e.g., *Chaetoceros decipiens* sp. 1 and 2) were present at the same site or during the same season in the molecular-based survey.

For both sites, greater species richness was recorded for the molecular-based (Fig. 3B, Mol) than morphology-based survey (Fig. 3B, Morph). At both sites, the majority (60% for PB and 80% for WV) of additional species recorded during the molecular-based survey (Fig. 3B, gray) were probably lumped into the general *Chaetoceros* spp. or *Thalassiosira* spp. categories during the morphology-based survey. Most of these taxa (67%) were common to both sites (i.e., *T. angulata*, *T. delicata*, *T. eccentrica*, and *T. pacifica*) and none of the taxa in this category were unique to WV. The additional species in this category at PB were *C. constrictus* and *T. antarctica*. The remaining additional taxa recorded in the molecular-based survey were due to cryptic species (Fig. 3B, vertical lines) with more found at PB (4) than at WV (1), most likely due to the higher abundance of *Chaetoceros* spp. at PB (Table 1). All of the taxa unique to the morphology-based survey (Fig. 3B, white) were in low abundance (i.e., fewer than five tow subsamples and never with a HELCOM rating>1; Table 1), except for *C. similis*, which was found in 18 tow subsamples, but with no HELCOM ratings>1 (Table 1).

When comparing seasons only winter, the season with the lowest species richness, had higher species richness recorded in the morphology-based (Fig. 3C, Morph) than the molecular-based survey (Fig. 3C, Mol). Taxa contributing to the higher species richness (i.e., *C. convolutus, C. debilis, C. furcellatus, C. laciniosus, C. similis*) were not abundant in winter (i.e., fewer than four tow subsamples, none with HELCOM ratings>1 in this season; Table 1) and were missed during the molecular-based survey with the techniques used here. The species that were unique to the molecular-based survey during winter were all *Thalassiosira* spp. (i.e., *T. delicata, T. eccentrica,* and *T. pacifica)*. The summer season had the highest species richness (Fig. 3C) and abundance (Table 1). None of the taxa missed by the molecular-based survey (i.e., *C. affinis*, *C. similis*, *T. nordenskioeldii*, and *Thalassiosira* tiny sp.) were abundant during this season (i.e., none with HELCOM ratings>1 in this season; Table 1). Taxa missed in the morphology-based survey were almost exclusively *Thalassiosira* spp. (as well as *C. constrictus*). The summer season had the greatest number of cryptic species pairs. The fall season had intermediate species richness (Fig. 3C) and all of the taxa missing from the molecular-based survey (i.e., *C. laciniosus, C. lorenzianus, C. similis,* and *T. baltica*) were again in low abundance (i.e., none with HELCOM ratings>1; Table 1). Species unique to the molecular-based survey in fall included: *C. diadema* sp. 1, *T. angulata, T. antarctica, T. eccentrica,* and *T. pacifica*. Two cryptic species pairs, *C. debilis* sp. 1 and 2 and *C. decipiens* sp. 1 and 2, were recorded during the molecular-based survey in this season.

## Discussion

### Similarities and Differences between the Morphology and Molecular-based Surveys

Half of the taxa (50%) were reported in the morphology and molecular-based surveys, indicating that both types of surveys provide a reasonably good estimation of the abundant, easily recognizable taxa. Therefore, monitoring efforts primarily concerned with abundant taxa can be confident using either approach, except where cryptic species are a concern. The majority (59%) of the matched species were *Chaetoceros* spp., possibly due to the way samples were processed for the morphology-based survey (i.e., live or formalin-acetic acid preserved samples). The features used for discriminating between *Chaetoceros* spp., such as setae position and length, shape and size of aperture between cells [38] are more easily discernable in live/recently preserved tow subsamples than the features used to identify *Thalassiosira* spp., such as position of labiate and strutted processes on the valve [38]. This difference in the ability to identify live material may have led to more lumping of individuals into the *Thalassiosira* spp. category than the *Chaetoceros* spp. category during the morphology-based survey and explain the bias against matches between morphology and molecular-based surveys for *Thalassiosira* spp. The high degree of similarity between morphology and molecular-based surveys in this study (50%) is in contrast to the 5% similarity reported by Savin et al. [3]. However, the differences in methodology between the two studies (i.e., whole water samples vs. plankton tows, denatured gradient gel electrophoresis (DGGE) vs. colony isolation, and the use of universal vs. diatom specific primers) likely explain the difference in congruence.

The other half of the taxa showed differences between the morphology and molecular-based surveys. The molecular-based survey excelled in identifying the six cryptic species pairs that were completely missed in the morphology-based surveys. In addition, by developing the DRL, the molecular-based survey was able to identify six *Thalassiosira* spp. that were most likely recorded simply as *Thalassiosira* spp. in the morphology-based survey due to the difficulty of their identification from live material. While more careful analysis during the morphology-based survey, such as cleaning the valves before identification and utilizing a scanning electron microscope (SEM), may increase the species-level identification of the *Thalassiosira* spp., the cryptic species pairs may remain difficult to distinguish without molecular data. Some of the cryptic *Chaetoceros* species pairs (e.g., *C. debilis, C. decipiens,* and *C. diadema*) did show seasonal trends indicating the possibility of different growth optima, but further research is necessary to determine whether these trends are consistent across multiple years with similar conditions. Many *Chaetoceros* spp. can form resting spores depending on conditions [46] so if the colonies are seasonal (as our data suggest), the ‘missing’ taxa are not necessarily absent, but may be surviving as resting spores until more favorable conditions are available.

The morphology-based survey excelled in identifying rare taxa. Of the taxa missed during the molecular-based survey, 80% were recorded in less than five tow subsamples in the morphology-based survey and none of them had HELCOM ratings>1 when they were missed. The molecular-based survey captured some rare taxa, but there was less consistency between the two methods when taxa were less abundant (i.e., either found in less than three tow subsamples or with a HELCOM rating = 1). One contributing factor to this discrepancy may be a result of comparing multiple subsamples – abundant taxa often show <10% variability between subsamples, but results for rare taxa are more variable [14]. In addition to this subsample variation, only ∼65% of the individual colonies isolated during the molecular-based survey resulted in positive amplification (most likely due to poor isolation or preservation since similar taxa amplified well during the DRL development). Therefore, rare taxa may have been isolated that did not amplify, leading to additional mismatch of rare taxa between methods. Unlike the previous factor, this problem may be eliminated through the use of next-generation sequencing technologies. However, this inconsistency between morphology and molecular-based surveys should not be dismissed. The rare taxa missed using either method could be important because different species may present different concerns to resource managers and/or cryptic species may go unnoticed and with potential economic consequences, especially if the unnoticed cryptic species produce toxins (e.g., some ‘cryptic’ *Pseudo-nitzschia* spp., [19]).

### Strengths and Limitations of Morphology and Molecular-based Surveys

The morphology-based survey was not able to identify the cryptic *Chaetoceros* spp. and also missed the less abundant (i.e., *T. angulata, T. antarctica, T. delicata*) or more difficult to identify (i.e., *T. eccentrica* and *T. pacifica*) *Thalassiosira* spp. However, the rare *Chaetoceros* spp. were more likely to be identified during the morphology-based survey, and missed in the molecular-based survey as discussed above. In addition to its ability to identify rare taxa, the morphology-based survey can more easily be adapted to producing quantitative abundance data (e.g., counting the number of cells in a specific volume). Using cleaned material for species identification and utilizing an SEM may improve the accuracy of the morphology-based survey, but this would also increase costs by adding materials (i.e., oxidizing chemicals, carbon tape, SEM stubs), time (i.e., time to process the materials, hourly rates for the SEM) and trained personnel (i.e., for processing samples and running the SEM). The equipment costs of the current morphology-based survey are lower than those for the molecular (i.e., identification books, slides, a microscope, etc.), but the personnel costs are high (i.e., time spent requiring a dedicated and highly trained taxonomic expert is critical for meaningful data acquisition).

The molecular-based survey was able to assign every specimen to a genetic species group, obviating the lumping of many individuals into general categories such as *Thalassiosira* spp. and *Chaetoceros* spp. In this study, genetic species groups were tied to species names, allowing even rare and cryptic taxa to be easily identified. Even in cases in which the sequence could not be tied to an identified species (e.g., *Thalassiosira* sp.), individual colonies could be assigned to genetic species groups and cataloged for future taxonomic determination. Also, the use of molecular data in continuing surveys would allow for comparisons between cryptic taxa over time considering abundance and seasonality of the different species. For example, *T. eccentrica* sp. 2 and *C. pseudobrevis* sp. 2 were only encountered in 2010 during the development of the DRL, and not during the 2011 molecular-based survey. This trend of different cryptic species being present/absent during different years would not be detected by the morphology-based survey.

However, development of the DRL and our molecular-based survey also have limitations. For instance, the isolation of colonies for developing the DRL was labor intensive and not all of the colonies isolated were successfully introduced into culture. However, the development of a DRL generates an ongoing and valuable resource. Indeed a majority (56%) of the species recorded in the morphology-based survey was found in our DRL after only one season of culture work. The DRL could be continually developed over subsequent years by targeting morphological species uncovered during the morphology and molecular-based surveys that are not yet represented in the database. After development of the DRL is complete, molecular technicians with limited taxonomic expertise could conduct the molecular-based surveys, a big advantage since the number of taxonomic experts is becoming more limited [47]. If technicians performed the routine surveys, this would also allow time for the taxonomic experts to critically validate the data generated, attempt to culture any new species observed, and pursue other research initiatives.

As for the limitations of the molecular-based survey, combining samples based on site and season may have missed trends in diatom abundance since methods are currently qualitative. Also, the molecular-based survey methodology used in this study was relatively costly requiring personnel with experience in isolating cells, various reagents, and extensive laboratory equipment (i.e., centrifuges, thermocycler, genetic analyzer, etc.). However, with decreasing sequencing costs [48], the limited number of taxonomic experts [47], and additional sequence data tied to morphologically identified specimens (e.g., www.barcodinglife.org and our DRL), the cost will be reduced relative to the enhanced benefits in the future. In addition, using new technologies such as next-generation sequencing to process environmental samples will improve the recovery of sequence data from rare taxa while decreasing the cost per sequence and personnel hours required to isolate colonies or cells making molecular-based surveys even more practical and affordable relative to the benefits. While the use of next-generation sequencing technology for qualitative comparisons is being used more frequently to investigate the diversity of microbial life, its use in determining quantitative abundance remains a work in progress due to PCR biases [49].

## Conclusions

The most effective means of measuring species richness for monitoring purposes will depend on the goals of a particular program. When species-level resolution is important, molecular-based surveys should be implemented or used to complement existing programs. In some cases, this resolution is unwarranted, but should be considered imperative for taxa that cause harmful algal blooms, contribute significantly to phytoplankton biomass, or are especially species-rich and/or difficult to identify. Our recommendation would be a combined approach with a continuation of routine morphology-based surveys and periodic molecular-based surveys to determine whether or not cryptic species are present, the seasonality of these cryptic species, and to record any non-indigenous cryptic species that may have been introduced to an area. This short-term study of only two diatom genera led to an increase in the known number of species in these two genera in the BoF by 25%. One of the labor-intensive portions of this study, developing the culture-based DRL, is not an ongoing expense since this legacy database has been established and can be augmented over the years by targeting morphological species known to harbor cryptic diversity based on the annual molecular-based surveys. With this publically available legacy database (www.barcodinglife.org), molecular-based surveys could become routine and less costly with improved sequence technology. While some researchers may conclude that matching sequence data to taxonomically validated species is too time consuming because the number of unique molecular sequences can provide rudimentary species richness, monitoring programs will continue to evaluate historic data (comparing the present to the past). Tying the sequence data of today and tomorrow to known and named species of the past will be, in our opinion, crucial for these studies.

## Supporting Information

Table S1 **Isolates sequenced from the Bay of Fundy in 2010–2011 that were utilized in this study.**
(DOCX)
