# Supplementary material for: A Comparison of Morphological and Molecular-Based Surveys to Estimate the Species Richness of Chaetoceros and Thalassiosira (Bacillariophyta), in the Bay of Fundy
Source: PLoS One. 2013 Oct 9;8(10):e73521. doi: 10.1371/journal.pone.0073521 (PMC3794052; doi:10.1371/journal.pone.0073521)
Supplement: Table S1 — Isolates sequenced from the Bay of Fundy in 2010–2011 that were utilized in this study. (DOCX) [file pone.0073521.s001.docx]

Table S1. Isolates sequenced from the Bay of Fundy in 2010-2011 that were utilized in this study.

| Taxon | Culture ID^a^ | BOLD ID^b^ | *rbc*L-3P | LSU D2/D3 | Site^c^ | Date/Season |
| --- | --- | --- | --- | --- | --- | --- |
| *Chaetoceros concavicornis* |  |  |  |  |  |  |
|  | - | BFMVM288-12 | KC985523 | - | WV | FALL |
| *Chaetoceros constrictus* |  |  |  |  |  |  |
|  | UNBF_P35A3 | TUBDI163-10 | KC985525 | KC986067 | PB | 15-Jun-10 |
|  | - | BFMVM235-12 | KC985524 | - | WV | SUMMER |
| *Chaetoceros contortus* sp. 1 |  |  |  |  |  |  |
|  | - | BFMVM030-12 | KC985526 | - | PB | SUMMER |
|  | - | BFMVM031-12 | KC985527 | - | PB | SUMMER |
| *Chaetoceros contortus* sp. 2 |  |  |  |  |  |  |
|  | - | BFMVM234-12 | KC985528 | - | WV | SUMMER |
| *Chaetoceros debilis* sp. 1 |  |  |  |  |  |  |
|  | UNBF_P17C3 | PDBOF057-11 | KC985587 | - | PB | 5-Apr-10 |
|  | UNBF_P32B5 | TUBDI172-10 | KC985582 | - | WV | 1-Jun-10 |
|  | - | BFMVM097-12 | KC985564 | - | PB | FALL |
|  | - | BFMVM106-12 | KC985548 | - | PB | FALL |
|  | - | BFMVM107-12 | KC985547 | - | PB | FALL |
|  | - | BFMVM108-12 | KC985546 | - | PB | FALL |
|  | - | BFMVM109-12 | KC985545 | - | PB | FALL |
|  | - | BFMVM110-12 | KC985544 | - | PB | FALL |
|  | - | BFMVM111-12 | KC985543 | - | PB | FALL |
|  | - | BFMVM112-12 | KC985542 | - | PB | FALL |
|  | - | BFMVM113-12 | KC985541 | - | PB | FALL |
|  | - | BFMVM114-12 | KC985539 | - | PB | FALL |
|  | - | BFMVM115-12 | KC985538 | - | PB | FALL |
|  | - | BFMVM098-12 | KC985561 | - | PB | FALL |
|  | - | BFMVM116-12 | KC985537 | - | PB | FALL |
|  | - | BFMVM117-12 | KC985530 | - | PB | FALL |
|  | - | BFMVM118-12 | KC985565 | - | PB | FALL |
|  | - | BFMVM119-12 | KC985552 | - | PB | FALL |
|  | - | BFMVM120-12 | KC985553 | - | PB | FALL |
|  | - | BFMVM121-12 | KC985566 | - | PB | FALL |
|  | - | BFMVM122-12 | KC985563 | - | PB | FALL |
|  | - | BFMVM123-12 | KC985567 | - | PB | FALL |
|  | - | BFMVM124-12 | KC985529 | - | PB | FALL |
|  | - | BFMVM125-12 | KC985555 | - | PB | FALL |
|  | - | BFMVM099-12 | KC985562 | - | PB | FALL |
|  | - | BFMVM126-12 | KC985554 | - | PB | FALL |
|  | - | BFMVM127-12 | KC985551 | - | PB | FALL |
|  | - | BFMVM128-12 | KC985550 | - | PB | FALL |
|  | - | BFMVM100-12 | KC985560 | - | PB | FALL |
|  | - | BFMVM101-12 | KC985559 | - | PB | FALL |
|  | - | BFMVM102-12 | KC985558 | - | PB | FALL |
|  | - | BFMVM103-12 | KC985557 | - | PB | FALL |
|  | - | BFMVM104-12 | KC985556 | - | PB | FALL |
|  | - | BFMVM105-12 | KC985549 | - | PB | FALL |
|  | - | BFMVM289-12 | KC985533 | - | WV | FALL |
|  | - | BFMVM290-12 | KC985534 | - | WV | FALL |
|  | - | BFMVM291-12 | KC985535 | - | WV | FALL |
|  | - | BFMVM292-12 | KC985536 | - | WV | FALL |
|  | - | BFMVM293-12 | KC985532 | - | WV | FALL |
| *Chaetoceros* *debilis* sp. 2 |  |  |  |  |  |  |
|  | UNBF_P80B3 | PDBOF037-11 | KC985540 | - | PB | 19-Oct-10 |
|  | UNBF_P9A1 | TUBDI054-10 | KC985531 | - | PB | 29-Jan-10 |
|  | - | BFMVM039-12 | KC985571 | - | PB | SUMMER |
|  | - | BFMVM040-12 | KC985572 | - | PB | SUMMER |
|  | - | BFMVM041-12 | KC985573 | - | PB | SUMMER |
|  | - | BFMVM042-12 | KC985574 | - | PB | SUMMER |
|  | - | BFMVM043-12 | KC985576 | - | PB | SUMMER |
|  | - | BFMVM044-12 | KC985577 | - | PB | SUMMER |
|  | - | BFMVM032-12 | KC985578 | - | PB | SUMMER |
|  | - | BFMVM033-12 | KC985575 | - | PB | SUMMER |
|  | - | BFMVM034-12 | KC985579 | - | PB | SUMMER |
|  | - | BFMVM035-12 | KC985583 | - | PB | SUMMER |
|  | - | BFMVM036-12 | KC985584 | - | PB | SUMMER |
|  | - | BFMVM037-12 | KC985569 | - | PB | SUMMER |
|  | - | BFMVM038-12 | KC985570 | - | PB | SUMMER |
|  | - | BFMVM129-12 | KC985585 | - | PB | FALL |
|  | - | BFMVM130-12 | KC985586 | - | PB | FALL |
|  | - | BFMVM236-12 | KC985568 | - | WV | SUMMER |
|  | - | BFMVM237-12 | KC985581 | - | WV | SUMMER |
|  | - | BFMVM238-12 | KC985580 | - | WV | SUMMER |
| *Chaetoceros decipiens* sp. 1 |  |  |  |  |  |  |
|  | UNBF_P67C4 | TUBDI257-10 | KC985591 | KC986068 | WV | 7-Sep-10 |
|  | - | BFMVM131-12 | KC985590 | - | PB | FALL |
|  | - | BFMVM132-12 | KC985588 | - | PB | FALL |
|  | - | BFMVM133-12 | KC985589 | - | PB | FALL |
|  | - | BFMVM134-12 | KC985592 | - | PB | FALL |
|  | - | BFMVM135-12 | KC985593 | - | PB | FALL |
| *Chaetoceros decipiens* sp. 2 |  |  |  |  |  |  |
|  | - | BFMVM001-12 | KC985601 | - | PB | WINTER |
|  | - | BFMVM045-12 | KC985600 | - | PB | SUMMER |
|  | - | BFMVM046-12 | KC985604 | - | PB | SUMMER |
|  | - | BFMVM047-12 | KC985596 | - | PB | SUMMER |
|  | - | BFMVM136-12 | KC985597 | - | PB | FALL |
|  | - | BFMVM196-12 | KC985603 | - | WV | WINTER |
|  | - | BFMVM197-12 | KC985602 | - | WV | WINTER |
|  | - | BFMVM297-12 | KC985605 | - | WV | FALL |
|  | - | BFMVM298-12 | KC985606 | - | WV | FALL |
|  | - | BFMVM299-12 | KC985599 | - | WV | FALL |
|  | - | BFMVM294-12 | KC985595 | - | WV | FALL |
|  | - | BFMVM295-12 | KC985594 | - | WV | FALL |
|  | - | BFMVM296-12 | KC985598 | - | WV | FALL |
| *Chaetoceros diadema* sp. 1 |  |  |  |  |  |  |
|  | UNBF_P38D5 | PDBOF118-11 | KC985623 | KC986073 | PB | 22-Jun-10 |
|  | UNBF_P62A2 | TUBDI230-10 | KC985618 | KC986071 | PB | 24-Aug-10 |
|  | UNBF_P68B2 | TUBDI258-10 | KC985615 | KC986069 | PB | 14-Sep-10 |
|  | UNBF_P68D1 | TUBDI261-10 | KC985616 | KC986070 | PB | 14-Sep-10 |
|  | UNBF_P73A5 | PDBOF022-11 | KC985617 | - | WV | 21-Sep-10 |
|  | UNBF_P81B1 | PDBOF039-11 | KC985621 | - | WV | 26-Oct-10 |
|  | UNBF_P82D2 | PDBOF044-11 | KC985619 | KC986072 | WV | 26-Oct-10 |
|  | UNBF_R38D5 | PDBOF111-11 | KC985622 | - | PB | 22-Jun-10 |
|  | - | BFMVM048-12 | KC985620 | - | PB | SUMMER |
|  | - | BFMVM049-12 | KC985609 | - | PB | SUMMER |
|  | - | BFMVM050-12 | KC985614 | - | PB | SUMMER |
|  | - | BFMVM051-12 | KC985613 | - | PB | SUMMER |
|  | - | BFMVM052-12 | KC985612 | - | PB | SUMMER |
|  | - | BFMVM053-12 | KC985611 | - | PB | SUMMER |
|  | - | BFMVM054-12 | KC985610 | - | PB | SUMMER |
|  | - | BFMVM137-12 | KC985624 | - | PB | FALL |
|  | - | BFMVM138-12 | KC985625 | - | PB | FALL |
|  | - | BFMVM139-12 | KC985607 | - | PB | FALL |
|  | - | BFMVM140-12 | KC985608 | - | PB | FALL |
| *Chaetoceros diadema* sp. 2 |  |  |  |  |  |  |
|  | UNBF_P10D2 | TUBDI064-10 | KC985640 | KC986079 | PB | 16-Feb-10 |
|  | UNBF_P18A3 | TUBDI101-10 | KC985636 | KC986077 | WV | 5-Apr-10 |
|  | UNBF_P20D1 | PDBOF067-11 | KC985629 | KC986075 | PB | 4-May-10 |
|  | UNBF_P21A4 | PDBOF116-11 | KC985627 | KC986074 | PB | 4-May-10 |
|  | UNBF_P36B3 | PDBOF074-11 | KC985630 | KC986076 | PB | 15-Jun-10 |
|  | UNBF_P44B2 | TUBDI198-10 | KC985639 | KC986078 | PB | 6-Jul-10 |
|  | - | BFMVM002-12 | KC985628 | - | PB | WINTER |
|  | - | BFMVM003-12 | KC985631 | - | PB | WINTER |
|  | - | BFMVM004-12 | KC985637 | - | PB | WINTER |
|  | - | BFMVM005-12 | KC985635 | - | PB | WINTER |
|  | - | BFMVM006-12 | KC985634 | - | PB | WINTER |
|  | - | BFMVM055-12 | KC985626 | - | PB | SUMMER |
|  | - | BFMVM056-12 | KC985638 | - | PB | SUMMER |
|  | - | BFMVM057-12 | KC985633 | - | PB | SUMMER |
|  | - | BFMVM058-12 | KC985632 | - | PB | SUMMER |
|  | - | BFMVM198-12 | KC985641 | - | WV | WINTER |
| *Chaetoceros didymus* |  |  |  |  |  |  |
|  | UNBF_P69D4 | PDBOF017-11 | KC985643 | KC986080 | WV | 14-Sep-10 |
|  | - | BFMVM059-12 | KC985644 | - | PB | SUMMER |
|  | - | BFMVM141-12 | KC985642 | - | PB | FALL |
| *Chaetoceros laciniosus* sp. 1 |  |  |  |  |  |  |
|  | UNBF_P20B5 | PDBOF065-11 | KC985648 | KC986083 | PB | 4-May-10 |
|  | UNBF_P20D3 | PDBOF068-11 | KC985647 | KC986082 | PB | 4-May-10 |
|  | UNBF_P55B5 | TUBDI212-10 | KC985645 | KC986081 | PB | 3-Aug-10 |
|  | - | BFMVM060-12 | KC985646 | - | PB | SUMMER |
| *Chaetoceros laciniosus* sp. 2 |  |  |  |  |  |  |
|  | - | BFMVM239-12 | KC985649 | - | WV | SUMMER |
| *Chaetoceros lorenzianus* sp. 1 |  |  |  |  |  |  |
|  | - | BFMVM061-12 | KC985650 | - | PB | SUMMER |
|  | - | BFMVM062-12 | KC985651 | - | PB | SUMMER |
| *Chaetoceros lorenzianus* sp. 2 |  |  |  |  |  |  |
|  | - | BFMVM063-12 | KC985652 | - | PB | SUMMER |
|  | - | BFMVM240-12 | KC985653 | - | WV | SUMMER |
| *Chaetoceros pseudobrevi*s sp. 1 | |  |  |  |  |  |
|  | UNBF_P31A1 | TUBDI147-10 | KC985654 | KC986084 | PB | 1-Jun-10 |
|  | UNBF_P36C5 | TUBDI166-10 | KC985655 | KC986085 | WV | 15-Jun-10 |
|  | - | BFMVM064-12 | KC985659 | - | PB | SUMMER |
|  | - | BFMVM065-12 | KC985658 | - | PB | SUMMER |
|  | - | BFMVM241-12 | KC985656 | - | WV | SUMMER |
|  | - | BFMVM242-12 | KC985657 | - | WV | SUMMER |
| *Chaetoceros pseudobrevis* sp. 2 | |  |  |  |  |  |
|  | UNBF_P52B2 | TUBDI209-10 | KC985660 | KC986086 | PB | 27-Jul-10 |
| *Chaetoceros radicans* sp. 1 |  |  |  |  |  |  |
|  | UNBF_P17D5 | TUBDI094-10 | KC985661 | KC986087 | PB | 5-Apr-10 |
| *Chaetoceros radicans* sp. 2 |  |  |  |  |  |  |
|  | - | BFMVM243-12 | KC985662 | - | WV | SUMMER |
| *Chaetoceros similis* |  |  |  |  |  |  |
|  | UNBF_P17B3 | TUBDI095-10 | KC985663 | KC986088 | PB | 5-Apr-10 |
| *Chaetoceros simplex* |  |  |  |  |  |  |
|  | UNBF_P40D3 | TUBDI192-10 | KC985664 | KC986089 | PB | 29-Jun-10 |
| *Chaetoceros socialis* |  |  |  |  |  |  |
|  | UNBF_P32D2 | TUBDI173-10 | KC985673 | - | PB | 1-Jun-10 |
|  | UNBF_P32D3 | TUBDI151-10 | KC985674 | KC986098 | PB | 1-Jun-10 |
|  | UNBF_P35A2 | TUBDI161-10 | KC985672 | KC986097 | PB | 15-Jun-10 |
|  | UNBF_P35C2 | PDBOF113-11 | KC985671 | KC986096 | PB | 15-Jun-10 |
|  | UNBF_P35C6 | TUBDI160-10 | KC985676 | KC986100 | PB | 15-Jun-10 |
|  | UNBF_P35D5 | TUBDI159-10 | - | KC986095 | PB | 15-Jun-10 |
|  | UNBF_P36A4 | TUBDI165-10 | KC985675 | KC986099 | PB | 15-Jun-10 |
|  | UNBF_P36A5 | TUBDI164-10 | KC985680 | KC986103 | PB | 15-Jun-10 |
|  | UNBF_P38C1 | PDBOF073-11 | KC985679 | KC986102 | PB | 22-Jun-10 |
|  | UNBF_P38D3 | PDBOF010-11 | KC985678 | - | PB | 22-Jun-10 |
|  | UNBF_P39B5 | PDBOF046-11 | KC985677 | KC986101 | WV | 22-Jun-10 |
|  | UNBF_P42A3 | TUBDI193-10 | KC985670 | KC986094 | WV | 29-Jun-10 |
|  | UNBF_P42C2 | TUBDI194-10 | KC985669 | - | WV | 29-Jun-10 |
|  | UNBF_P42C6 | TUBDI196-10 | KC985668 | KC986093 | WV | 29-Jun-10 |
|  | UNBF_P43A5 | TUBDI197-10 | KC985667 | KC986092 | PB | 6-Jul-10 |
|  | UNBF_P45C6 | TUBDI200-10 | KC985666 | KC986091 | WV | 6-Jul-10 |
|  | UNBF_P46B6 | PDBOF011-11 | KC985665 | KC986090 | PB | 13-Jul-10 |
|  | UNBF_P48A1 | TUBDI202-10 | KC985683 | KC986105 | WV | 13-Jul-10 |
|  | UNBF_P48D2 | TUBDI203-10 | KC985681 | KC986104 | WV | 13-Jul-10 |
|  | UNBF_P52C2 | PDBOF013-11 | KC985682 | - | PB | 27-Jul-10 |
| *Chaetoceros teres* |  |  |  |  |  |  |
|  | UNBF_P20D4 | PDBOF069-11 | KC985684 | KC986106 | PB | 4-May-10 |
| *Thalassiosira angulata* |  |  |  |  |  |  |
|  | UNBF_P30C2 | TUBDI145-10 | KC985691 | KC986109 | PB | 1-Jun-10 |
|  | UNBF_P40B1 | TUBDI190-10 | KC985686 | KC986107 | PB | 29-Jun-10 |
|  | UNBF_P65A5 | PDBOF015-11 | KC985687 | KC986108 | PB | 7-Sep-10 |
|  | - | BFMVM142-12 | KC985688 | - | PB | FALL |
|  | - | BFMVM244-12 | KC985692 | - | WV | SUMMER |
|  | - | BFMVM245-12 | KC985693 | - | WV | SUMMER |
|  | - | BFMVM246-12 | KC985694 | - | WV | SUMMER |
|  | - | BFMVM247-12 | KC985695 | - | WV | SUMMER |
|  | - | BFMVM300-12 | KC985690 | - | WV | FALL |
|  | - | BFMVM301-12 | KC985689 | - | WV | FALL |
| *Thalassiosira anguste-lineata* |  |  |  |  |  |  |
|  | UNBF_P19B5 | TUBDI110-10 | KC985698 | KC986110 | WV | 5-Apr-10 |
|  | UNBF_P33D2 | TUBDI155-10 | KC985705 | KC986115 | PB | 8-Jun-10 |
|  | UNBF_P45A5 | TUBDI199-10 | KC985701 | KC986112 | WV | 6-Jul-10 |
|  | UNBF_P62B4 | TUBDI234-10 | KC985703 | KC986113 | PB | 24-Aug-10 |
|  | UNBF_P63C4 | TUBDI236-10 | KC985704 | KC986114 | WV | 24-Aug-10 |
|  | UNBF_P78C4 | PDBOF033-11 | KC985719 | KC986117 | WV | 12-Oct-10 |
|  | UNBF_P86C3 | PDBOF097-11 | KC985708 | KC986116 | WV | 16-Nov-10 |
|  | UNBF_P8D3 | TUBDI035-10 | KC985700 | KC986111 | PB | 27-Jan-10 |
|  | UNBF_P9C1 | TUBDI055-10 | KC985716 | - | PB | 29-Jan-10 |
|  | - | BFMVM010-12 | KC985718 | - | PB | WINTER |
|  | - | BFMVM011-12 | KC985709 | - | PB | WINTER |
|  | - | BFMVM007-12 | KC985710 | - | PB | WINTER |
|  | - | BFMVM008-12 | KC985706 | - | PB | WINTER |
|  | - | BFMVM009-12 | KC985707 | - | PB | WINTER |
|  | - | BFMVM143-12 | KC985702 | - | PB | FALL |
|  | - | BFMVM199-12 | KC985714 | - | WV | WINTER |
|  | - | BFMVM200-12 | KC985713 | - | WV | WINTER |
|  | - | BFMVM201-12 | KC985712 | - | WV | WINTER |
|  | - | BFMVM202-12 | KC985711 | - | WV | WINTER |
|  | - | BFMVM248-12 | KC985720 | - | WV | SUMMER |
|  | - | BFMVM302-12 | KC985715 | - | WV | FALL |
|  | - | BFMVM303-12 | KC985717 | - | WV | FALL |
|  | - | BFMVM304-12 | KC985696 | - | WV | FALL |
|  | - | BFMVM305-12 | KC985697 | - | WV | FALL |
|  | - | BFMVM306-12 | KC985699 | - | WV | FALL |
| *Thalassiosira antarctica* |  |  |  |  |  |  |
|  | UNBF_P10D5 | TUBDI088-10 | - | KC986119 | PB | 16-Feb-10 |
|  | UNBF_P67B2 | TUBDI253-10 | KC985729 | KC986120 | WV | 7-Sep-10 |
|  | UNBF_P8A2 | TUBDI029-10 | KC985723 | KC986118 | PB | 27-Jan-10 |
|  | - | BFMVM249-12 | KC985724 | - | WV | SUMMER |
|  | - | BFMVM250-12 | KC985725 | - | WV | SUMMER |
|  | - | BFMVM251-12 | KC985721 | - | WV | SUMMER |
|  | - | BFMVM252-12 | KC985726 | - | WV | SUMMER |
|  | - | BFMVM253-12 | KC985727 | - | WV | SUMMER |
|  | - | BFMVM254-12 | KC985722 | - | WV | SUMMER |
|  | - | BFMVM307-12 | KC985728 | - | WV | FALL |
| *Thalassiosira baltica* |  |  |  |  |  |  |
|  | - | BFMVM029-12 | KC985731 | - | PB | WINTER |
|  | - | BFMVM287-12 | KC985730 | - | WV | SUMMER |
| *Thalassiosira bioculata* var. *exigua* | |  |  |  |  |  |
|  | - | BFMVM308-12 | KC985733 | - | WV | FALL |
|  | - | BFMVM309-12 | KC985732 | - | WV | FALL |
| *Thalassiosira decipiens* |  |  |  |  |  |  |
|  | UNBF_P10D1 | TUBDI089-10 | KC985735 | KC986122 | PB | 16-Feb-10 |
|  | UNBF_P11D1 | TUBDI069-10 | KC985734 | KC986121 | PB | 16-Feb-10 |
| *Thalassiosira delicata* |  |  |  |  |  |  |
|  | UNBF_P20C2 | PDBOF066-11 | KC985737 | KC986123 | PB | 4-May-10 |
|  | UNBF_P34C3 | PDBOF009-11 | KC985738 | KC986124 | WV | 8-Jun-10 |
|  | - | BFMVM203-12 | KC985736 | - | WV | WINTER |
|  | - | BFMVM255-12 | KC985739 | - | WV | SUMMER |
| *Thalassiosira eccentrica* sp. 1 |  |  |  |  |  |  |
|  | UNBF_P34B4 | TUBDI177-10 | KC985749 | - | WV | 8-Jun-10 |
|  | UNBF_P34B5 | TUBDI158-10 | KC985748 | KC986128 | WV | 8-Jun-10 |
|  | UNBF_P37B2 | TUBDI176-10 | KC985747 | - | WV | 15-Jun-10 |
|  | UNBF_P37B6 | TUBDI178-10 | KC985746 | - | WV | 15-Jun-10 |
|  | UNBF_P38B5 | TUBDI186-10 | KC985745 | KC986127 | PB | 22-Jun-10 |
|  | UNBF_P39D5 | TUBDI189-10 | KC985744 | - | WV | 22-Jun-10 |
|  | UNBF_P47A1 | TUBDI201-10 | KC985743 | KC986126 | PB | 13-Jul-10 |
|  | UNBF_P49D4 | TUBDI206-10 | KC985742 | - | PB | 20-Jul-10 |
|  | UNBF_P55C6 | TUBDI213-10 | KC985741 | - | PB | 3-Aug-10 |
|  | UNBF_P66C1 | TUBDI245-10 | KC985740 | KC986125 | PB | 7-Sep-10 |
|  | UNBF_P67B6 | TUBDI256-10 | KC985763 | KC986136 | WV | 7-Sep-10 |
|  | UNBF_P75B1 | PDBOF026-11 | KC985762 | KC986135 | WV | 28-Sep-10 |
|  | UNBF_P76B2 | PDBOF029-11 | KC985761 | KC986134 | PB | 5-Oct-10 |
|  | UNBF_P82A3 | PDBOF042-11 | KC985760 | KC986133 | PB | 26-Oct-10 |
|  | UNBF_P84D5 | PDBOF088-11 | KC985759 | KC986132 | PB | 16-Nov-10 |
|  | UNBF_P85D2 | PDBOF093-11 | KC985758 | KC986131 | WV | 16-Nov-10 |
|  | UNBF_P88B4 | PDBOF106-11 | KC985757 | KC986130 | WV | 14-Dec-10 |
|  | UNBF_R55B4 | PDBOF110-11 | KC985756 | KC986129 | PB | 3-Aug-10 |
|  | - | BFMVM012-12 | KC985750 | - | PB | WINTER |
|  | - | BFMVM144-12 | KC985770 | - | PB | FALL |
|  | - | BFMVM145-12 | KC985769 | - | PB | FALL |
|  | - | BFMVM146-12 | KC985768 | - | PB | FALL |
|  | - | BFMVM147-12 | KC985767 | - | PB | FALL |
|  | - | BFMVM148-12 | KC985766 | - | PB | FALL |
|  | - | BFMVM149-12 | KC985765 | - | PB | FALL |
|  | - | BFMVM150-12 | KC985764 | - | PB | FALL |
|  | - | BFMVM205-12 | KC985754 | - | WV | WINTER |
|  | - | BFMVM206-12 | KC985753 | - | WV | WINTER |
|  | - | BFMVM207-12 | KC985752 | - | WV | WINTER |
|  | - | BFMVM204-12 | KC985755 | - | WV | WINTER |
|  | - | BFMVM256-12 | KC985751 | - | WV | SUMMER |
|  | - | BFMVM310-12 | KC985774 | - | WV | FALL |
|  | - | BFMVM311-12 | KC985773 | - | WV | FALL |
|  | - | BFMVM312-12 | KC985772 | - | WV | FALL |
|  | - | BFMVM313-12 | KC985771 | - | WV | FALL |
| *Thalassiosira eccentrica* sp. 2 |  |  |  |  |  |  |
|  | UNBF_P56B3 | TUBDI214-10 | KC985775 | KC986137 | WV | 3-Aug-10 |
|  | UNBF_P58A1 | TUBDI217-10 | KC985777 | KC986139 | WV | 10-Aug-10 |
|  | UNBF_P58D6 | TUBDI222-10 | KC985779 | KC986141 | WV | 10-Aug-10 |
|  | UNBF_P62A3 | TUBDI231-10 | KC985776 | KC986138 | PB | 24-Aug-10 |
|  | UNBF_P62B1 | TUBDI233-10 | KC985783 | KC986145 | PB | 24-Aug-10 |
|  | UNBF_P65A6 | TUBDI241-10 | KC985781 | KC986143 | PB | 7-Sep-10 |
|  | UNBF_P66A1 | TUBDI242-10 | KC985782 | KC986144 | PB | 7-Sep-10 |
|  | UNBF_P66C5 | TUBDI247-10 | KC985778 | KC986140 | PB | 7-Sep-10 |
|  | UNBF_P84A2 | PDBOF084-11 | KC985780 | KC986142 | PB | 16-Nov-10 |
| *Thalassiosira gravida* |  |  |  |  |  |  |
|  | UNBF_P11C6 | TUBDI068-10 | KC985785 | KC986146 | PB | 16-Feb-10 |
|  | UNBF_P13D5 | TUBDI076-10 | KC985835 | - | PB | 16-Mar-10 |
|  | UNBF_P14B2 | TUBDI078-10 | KC985834 | KC986158 | PB | 16-Mar-10 |
|  | UNBF_P14B5 | TUBDI080-10 | KC985833 | KC986157 | PB | 16-Mar-10 |
|  | UNBF_P23D5 | TUBDI119-10 | KC985799 | KC986154 | PB | 4-May-10 |
|  | UNBF_P49D5 | TUBDI207-10 | KC985797 | KC986153 | PB | 20-Jul-10 |
|  | UNBF_P58A6 | TUBDI218-10 | KC985829 | KC986156 | WV | 10-Aug-10 |
|  | UNBF_P59B6 | TUBDI224-10 | - | KC986155 | PB | 17-Aug-10 |
|  | UNBF_P7A3 | TUBDI023-10 | KC985838 | KC986159 | PB | 27-Jan-10 |
|  | UNBF_P7B3 | TUBDI025-10 | KC985839 | KC986160 | PB | 27-Jan-10 |
|  | UNBF_P84B3 | PDBOF087-11 | KC985786 | KC986147 | PB | 16-Nov-10 |
|  | UNBF_P85D3 | PDBOF094-11 | KC985787 | KC986148 | WV | 16-Nov-10 |
|  | UNBF_P88D2 | PDBOF108-11 | KC985788 | KC986149 | WV | 14-Dec-10 |
|  | UNBF_P8B3 | TUBDI031-10 | KC985789 | KC986150 | PB | 27-Jan-10 |
|  | UNBF_P8C2 | TUBDI033-10 | KC985790 | KC986151 | PB | 27-Jan-10 |
|  | UNBF_P8C3 | TUBDI034-10 | KC985791 | KC986152 | PB | 27-Jan-10 |
|  | - | BFMVM066-12 | KC985822 | - | PB | SUMMER |
|  | - | BFMVM067-12 | KC985823 | - | PB | SUMMER |
|  | - | BFMVM068-12 | KC985824 | - | PB | SUMMER |
|  | - | BFMVM151-12 | KC985809 | - | PB | FALL |
|  | - | BFMVM152-12 | KC985810 | - | PB | FALL |
|  | - | BFMVM153-12 | KC985811 | - | PB | FALL |
|  | - | BFMVM154-12 | KC985830 | - | PB | FALL |
|  | - | BFMVM208-12 | KC985828 | - | WV | WINTER |
|  | - | BFMVM209-12 | KC985827 | - | WV | WINTER |
|  | - | BFMVM257-12 | KC985819 | - | WV | SUMMER |
|  | - | BFMVM258-12 | KC985818 | - | WV | SUMMER |
|  | - | BFMVM259-12 | KC985817 | - | WV | SUMMER |
|  | - | BFMVM260-12 | KC985816 | - | WV | SUMMER |
|  | - | BFMVM261-12 | KC985815 | - | WV | SUMMER |
|  | - | BFMVM262-12 | KC985814 | - | WV | SUMMER |
|  | - | BFMVM263-12 | KC985813 | - | WV | SUMMER |
|  | - | BFMVM264-12 | KC985831 | - | WV | SUMMER |
|  | - | BFMVM265-12 | KC985812 | - | WV | SUMMER |
|  | - | BFMVM266-12 | KC985798 | - | WV | SUMMER |
|  | - | BFMVM267-12 | KC985820 | - | WV | SUMMER |
|  | - | BFMVM268-12 | KC985821 | - | WV | SUMMER |
|  | - | BFMVM269-12 | KC985825 | - | WV | SUMMER |
|  | - | BFMVM270-12 | KC985826 | - | WV | SUMMER |
|  | - | BFMVM271-12 | KC985792 | - | WV | SUMMER |
|  | - | BFMVM272-12 | KC985793 | - | WV | SUMMER |
|  | - | BFMVM273-12 | KC985794 | - | WV | SUMMER |
|  | - | BFMVM274-12 | KC985795 | - | WV | SUMMER |
|  | - | BFMVM275-12 | KC985796 | - | WV | SUMMER |
|  | - | BFMVM314-12 | KC985804 | - | WV | FALL |
|  | - | BFMVM315-12 | KC985805 | - | WV | FALL |
|  | - | BFMVM316-12 | KC985806 | - | WV | FALL |
|  | - | BFMVM317-12 | KC985807 | - | WV | FALL |
|  | - | BFMVM318-12 | KC985808 | - | WV | FALL |
|  | - | BFMVM319-12 | KC985832 | - | WV | FALL |
|  | - | BFMVM320-12 | KC985836 | - | WV | FALL |
|  | - | BFMVM321-12 | KC985837 | - | WV | FALL |
|  | - | BFMVM322-12 | KC985784 | - | WV | FALL |
|  | - | BFMVM323-12 | KC985800 | - | WV | FALL |
|  | - | BFMVM324-12 | KC985801 | - | WV | FALL |
|  | - | BFMVM325-12 | KC985802 | - | WV | FALL |
|  | - | BFMVM326-12 | KC985803 | - | WV | FALL |
| *Thalassiosira nordenskioeldii* |  |  |  |  |  |  |
|  | UNBF_P10B5 | TUBDI059-10 | KC985862 | KC986165 | PB | 16-Feb-10 |
|  | UNBF_P12B1 | TUBDI072-10 | KC985863 | KC986166 | PB | 16-Feb-10 |
|  | UNBF_P12B4 | TUBDI073-10 | KC985864 | KC986167 | PB | 16-Feb-10 |
|  | UNBF_P17C4 | TUBDI100-10 | KC985865 | KC986168 | PB | 5-Apr-10 |
|  | UNBF_P18A6 | TUBDI106-10 | KC985866 | KC986169 | WV | 5-Apr-10 |
|  | UNBF_P18B1 | TUBDI107-10 | KC985867 | KC986170 | WV | 5-Apr-10 |
|  | UNBF_P18C1 | TUBDI182-10 | KC985868 | - | WV | 5-Apr-10 |
|  | UNBF_P19A1 | PDBOF061-11 | KC985869 | KC986171 | WV | 5-Apr-10 |
|  | UNBF_P19A6 | PDBOF062-11 | KC985870 | KC986172 | WV | 5-Apr-10 |
|  | UNBF_P20A2 | PDBOF064-11 | KC985871 | KC986173 | PB | 4-May-10 |
|  | UNBF_P20D5 | TUBDI112-10 | KC985872 | KC986174 | PB | 4-May-10 |
|  | UNBF_P23B3 | TUBDI121-10 | - | KC986175 | PB | 4-May-10 |
|  | UNBF_P23C1 | TUBDI122-10 | KC985873 | KC986176 | PB | 4-May-10 |
|  | UNBF_P23D3 | TUBDI123-10 | KC985874 | KC986177 | PB | 4-May-10 |
|  | UNBF_P24C4 | TUBDI125-10 | KC985875 | KC986178 | PB | 12-May-10 |
|  | UNBF_P24D5 | TUBDI126-10 | KC985876 | KC986179 | PB | 12-May-10 |
|  | UNBF_P24D6 | TUBDI183-10 | KC985840 | - | PB | 12-May-10 |
|  | UNBF_P29A3 | TUBDI184-10 | KC985877 | - | WV | 18-May-10 |
|  | UNBF_P30C5 | TUBDI146-10 | KC985878 | KC986180 | PB | 1-Jun-10 |
|  | UNBF_P9A2 | TUBDI036-10 | KC985861 | KC986164 | PB | 29-Jan-10 |
|  | UNBF_P9B2 | TUBDI037-10 | KC985860 | KC986163 | PB | 29-Jan-10 |
|  | UNBF_P9B5 | TUBDI038-10 | KC985859 | KC986162 | PB | 29-Jan-10 |
|  | UNBF_P9D6 | TUBDI040-10 | KC985858 | KC986161 | PB | 29-Jan-10 |
|  | - | BFMVM013-12 | KC985879 | - | PB | WINTER |
|  | - | BFMVM014-12 | KC985880 | - | PB | WINTER |
|  | - | BFMVM015-12 | KC985881 | - | PB | WINTER |
|  | - | BFMVM016-12 | KC985882 | - | PB | WINTER |
|  | - | BFMVM017-12 | KC985883 | - | PB | WINTER |
|  | - | BFMVM018-12 | KC985884 | - | PB | WINTER |
|  | - | BFMVM210-12 | KC985857 | - | WV | WINTER |
|  | - | BFMVM211-12 | KC985856 | - | WV | WINTER |
|  | - | BFMVM212-12 | KC985855 | - | WV | WINTER |
|  | - | BFMVM213-12 | KC985854 | - | WV | WINTER |
|  | - | BFMVM214-12 | KC985853 | - | WV | WINTER |
|  | - | BFMVM215-12 | KC985852 | - | WV | WINTER |
|  | - | BFMVM216-12 | KC985851 | - | WV | WINTER |
|  | - | BFMVM217-12 | KC985850 | - | WV | WINTER |
|  | - | BFMVM218-12 | KC985849 | - | WV | WINTER |
|  | - | BFMVM219-12 | KC985848 | - | WV | WINTER |
|  | - | BFMVM220-12 | KC985847 | - | WV | WINTER |
|  | - | BFMVM221-12 | KC985846 | - | WV | WINTER |
|  | - | BFMVM222-12 | KC985845 | - | WV | WINTER |
|  | - | BFMVM223-12 | KC985844 | - | WV | WINTER |
|  | - | BFMVM224-12 | KC985843 | - | WV | WINTER |
|  | - | BFMVM225-12 | KC985842 | - | WV | WINTER |
|  | - | BFMVM226-12 | KC985841 | - | WV | WINTER |
| *Thalassiosira pacifica* |  |  |  |  |  |  |
|  | UNBF_P10B6 | TUBDI060-10 | KC985886 | KC986182 | PB | 16-Feb-10 |
|  | UNBF_P13D6 | PDBOF001-11 | KC985893 | KC986183 | PB | 16-Mar-10 |
|  | UNBF_P14D1 | TUBDI083-10 | KC985901 | KC986184 | PB | 16-Mar-10 |
|  | UNBF_P15B5 | PDBOF055-11 | KC985910 | KC986185 | PB | 16-Mar-10 |
|  | UNBF_P17A1 | PDBOF002-11 | KC985911 | KC986186 | PB | 5-Apr-10 |
|  | UNBF_P17A5 | TUBDI098-10 | - | KC986187 | PB | 5-Apr-10 |
|  | UNBF_P17B1 | PDBOF003-11 | KC985912 | KC986188 | PB | 5-Apr-10 |
|  | UNBF_P17B5 | PDBOF004-11 | KC985913 | KC986189 | PB | 5-Apr-10 |
|  | UNBF_P17C6 | TUBDI099-10 | KC985915 | KC986190 | PB | 5-Apr-10 |
|  | UNBF_P17D1 | TUBDI097-10 | KC985916 | KC986191 | PB | 5-Apr-10 |
|  | UNBF_P18A1 | TUBDI103-10 | KC985917 | KC986192 | WV | 5-Apr-10 |
|  | UNBF_P18A2 | TUBDI104-10 | KC985918 | KC986193 | WV | 5-Apr-10 |
|  | UNBF_P18A4 | TUBDI105-10 | - | KC986194 | WV | 5-Apr-10 |
|  | UNBF_P18B2 | PDBOF058-11 | KC985921 | KC986195 | WV | 5-Apr-10 |
|  | UNBF_P18B3 | PDBOF059-11 | KC985922 | KC986196 | WV | 5-Apr-10 |
|  | UNBF_P18D1 | PDBOF114-11 | KC985924 | KC986197 | WV | 5-Apr-10 |
|  | UNBF_P19B1 | PDBOF063-11 | KC985927 | KC986198 | WV | 5-Apr-10 |
|  | UNBF_P19B2 | TUBDI111-10 | KC985934 | KC986199 | WV | 5-Apr-10 |
|  | UNBF_P19B4 | PDBOF005-11 | KC985935 | - | WV | 5-Apr-10 |
|  | UNBF_P22B2 | TUBDI118-10 | KC985938 | KC986200 | WV | 4-May-10 |
|  | UNBF_P22B5 | PDBOF006-11 | KC985939 | KC986201 | WV | 4-May-10 |
|  | UNBF_P22C5 | TUBDI117-10 | KC985885 | KC986181 | WV | 4-May-10 |
|  | UNBF_P22D1 | TUBDI116-10 | KC985940 | KC986202 | WV | 4-May-10 |
|  | UNBF_P23A3 | TUBDI120-10 | KC985941 | KC986203 | WV | 4-May-10 |
|  | UNBF_P25B2 | TUBDI129-10 | KC985949 | KC986204 | WV | 12-May-10 |
|  | UNBF_P28A2 | TUBDI141-10 | KC985950 | KC986205 | PB | 18-May-10 |
|  | UNBF_P31D6 | TUBDI150-10 | KC985960 | KC986207 | WV | 1-Jun-10 |
|  | UNBF_P9C6 | TUBDI170-10 | KC985961 | KC986208 | PB | 29-Jan-10 |
|  | UNBF_R14D2 | PDBOF109-11 | KC985959 | KC986206 | PB | 16-Mar-10 |
|  | - | BFMVM019-12 | KC985923 | - | PB | WINTER |
|  | - | BFMVM020-12 | KC985925 | - | PB | WINTER |
|  | - | BFMVM021-12 | KC985926 | - | PB | WINTER |
|  | - | BFMVM022-12 | KC985920 | - | PB | WINTER |
|  | - | BFMVM023-12 | KC985919 | - | PB | WINTER |
|  | - | BFMVM024-12 | KC985889 | - | PB | WINTER |
|  | - | BFMVM025-12 | KC985888 | - | PB | WINTER |
|  | - | BFMVM026-12 | KC985887 | - | PB | WINTER |
|  | - | BFMVM027-12 | KC985957 | - | PB | WINTER |
|  | - | BFMVM069-12 | KC985914 | - | PB | SUMMER |
|  | - | BFMVM070-12 | KC985891 | - | PB | SUMMER |
|  | - | BFMVM071-12 | KC985890 | - | PB | SUMMER |
|  | - | BFMVM072-12 | KC985964 | - | PB | SUMMER |
|  | - | BFMVM073-12 | KC985952 | - | PB | SUMMER |
|  | - | BFMVM074-12 | KC985951 | - | PB | SUMMER |
|  | - | BFMVM075-12 | KC985947 | - | PB | SUMMER |
|  | - | BFMVM076-12 | KC985963 | - | PB | SUMMER |
|  | - | BFMVM077-12 | KC985948 | - | PB | SUMMER |
|  | - | BFMVM078-12 | KC985945 | - | PB | SUMMER |
|  | - | BFMVM079-12 | KC985933 | - | PB | SUMMER |
|  | - | BFMVM080-12 | KC985932 | - | PB | SUMMER |
|  | - | BFMVM081-12 | KC985931 | - | PB | SUMMER |
|  | - | BFMVM082-12 | KC985930 | - | PB | SUMMER |
|  | - | BFMVM083-12 | KC985909 | - | PB | SUMMER |
|  | - | BFMVM084-12 | KC985908 | - | PB | SUMMER |
|  | - | BFMVM085-12 | KC985907 | - | PB | SUMMER |
|  | - | BFMVM086-12 | KC985900 | - | PB | SUMMER |
|  | - | BFMVM087-12 | KC985899 | - | PB | SUMMER |
|  | - | BFMVM088-12 | KC985898 | - | PB | SUMMER |
|  | - | BFMVM089-12 | KC985897 | - | PB | SUMMER |
|  | - | BFMVM090-12 | KC985896 | - | PB | SUMMER |
|  | - | BFMVM091-12 | KC985895 | - | PB | SUMMER |
|  | - | BFMVM092-12 | KC985894 | - | PB | SUMMER |
|  | - | BFMVM093-12 | KC985892 | - | PB | SUMMER |
|  | - | BFMVM094-12 | KC985958 | - | PB | SUMMER |
|  | - | BFMVM095-12 | KC985962 | - | PB | SUMMER |
|  | - | BFMVM227-12 | KC985902 | - | WV | WINTER |
|  | - | BFMVM228-12 | KC985903 | - | WV | WINTER |
|  | - | BFMVM229-12 | KC985904 | - | WV | WINTER |
|  | - | BFMVM230-12 | KC985905 | - | WV | WINTER |
|  | - | BFMVM231-12 | KC985906 | - | WV | WINTER |
|  | - | BFMVM276-12 | KC985946 | - | WV | SUMMER |
|  | - | BFMVM277-12 | KC985944 | - | WV | SUMMER |
|  | - | BFMVM278-12 | KC985943 | - | WV | SUMMER |
|  | - | BFMVM279-12 | KC985942 | - | WV | SUMMER |
|  | - | BFMVM280-12 | KC985937 | - | WV | SUMMER |
|  | - | BFMVM281-12 | KC985936 | - | WV | SUMMER |
|  | - | BFMVM282-12 | KC985956 | - | WV | SUMMER |
|  | - | BFMVM283-12 | KC985955 | - | WV | SUMMER |
|  | - | BFMVM284-12 | KC985954 | - | WV | SUMMER |
|  | - | BFMVM285-12 | KC985953 | - | WV | SUMMER |
|  | - | BFMVM327-12 | KC985929 | - | WV | FALL |
|  | - | BFMVM328-12 | KC985928 | - | WV | FALL |
| *Thalassiosira punctigera* |  |  |  |  |  |  |
|  | UNBF_P10D4 | TUBDI179-10 | KC985995 | KC986213 | PB | 16-Feb-10 |
|  | UNBF_P11D4 | TUBDI070-10 | KC986011 | KC986222 | PB | 16-Feb-10 |
|  | UNBF_P14A5 | TUBDI077-10 | KC986026 | KC986229 | PB | 16-Mar-10 |
|  | UNBF_P14B6 | TUBDI081-10 | KC986030 | KC986230 | PB | 16-Mar-10 |
|  | UNBF_P14D2 | TUBDI084-10 | KC986036 | - | PB | 16-Mar-10 |
|  | UNBF_P14D5 | TUBDI085-10 | KC986037 | KC986231 | PB | 16-Mar-10 |
|  | UNBF_P19C4 | TUBDI109-10 | KC986055 | KC986232 | WV | 5-Apr-10 |
|  | UNBF_P32A3 | TUBDI153-10 | KC986063 | KC986233 | WV | 1-Jun-10 |
|  | UNBF_P37A3 | TUBDI169-10 | KC986064 | KC986234 | WV | 15-Jun-10 |
|  | UNBF_P74A4 | PDBOF023-11 | KC986023 | KC986228 | PB | 28-Sep-10 |
|  | UNBF_P74B5 | PDBOF024-11 | KC986022 | KC986227 | PB | 28-Sep-10 |
|  | UNBF_P76B1 | PDBOF028-11 | KC986020 | KC986226 | PB | 5-Oct-10 |
|  | UNBF_P78A1 | PDBOF030-11 | KC986018 | KC986225 | PB | 12-Oct-10 |
|  | UNBF_P7B4 | TUBDI026-10 | KC986014 | KC986224 | PB | 27-Jan-10 |
|  | UNBF_P80A3 | PDBOF036-11 | KC986013 | KC986223 | PB | 19-Oct-10 |
|  | UNBF_P81D4 | PDBOF040-11 | KC986010 | KC986221 | PB | 26-Oct-10 |
|  | UNBF_P82A2 | PDBOF041-11 | KC986009 | KC986220 | PB | 26-Oct-10 |
|  | UNBF_P82D1 | PDBOF043-11 | KC986007 | KC986219 | WV | 26-Oct-10 |
|  | UNBF_P84A1 | PDBOF083-11 | KC986005 | KC986218 | PB | 16-Nov-10 |
|  | UNBF_P84A3 | PDBOF085-11 | KC986003 | KC986217 | PB | 16-Nov-10 |
|  | UNBF_P84B1 | PDBOF086-11 | KC986002 | KC986216 | PB | 16-Nov-10 |
|  | UNBF_P85B1 | PDBOF089-11 | KC985999 | KC986215 | PB | 16-Nov-10 |
|  | UNBF_P86C1 | PDBOF096-11 | KC985996 | KC986214 | WV | 16-Nov-10 |
|  | UNBF_P87A5 | PDBOF100-11 | KC985994 | KC986212 | PB | 14-Dec-10 |
|  | UNBF_P87C2 | PDBOF101-11 | KC985993 | KC986211 | PB | 14-Dec-10 |
|  | UNBF_P87C5 | PDBOF102-11 | KC985992 | KC986210 | PB | 14-Dec-10 |
|  | UNBF_P8B4 | TUBDI032-10 | KC985987 | KC986209 | PB | 27-Jan-10 |
|  | - | BFMVM028-12 | KC986049 | - | PB | WINTER |
|  | - | BFMVM096-12 | KC985965 | - | PB | SUMMER |
|  | - | BFMVM155-12 | KC986048 | - | PB | FALL |
|  | - | BFMVM156-12 | KC986047 | - | PB | FALL |
|  | - | BFMVM157-12 | KC986046 | - | PB | FALL |
|  | - | BFMVM158-12 | KC986045 | - | PB | FALL |
|  | - | BFMVM159-12 | KC986044 | - | PB | FALL |
|  | - | BFMVM160-12 | KC986043 | - | PB | FALL |
|  | - | BFMVM161-12 | KC986042 | - | PB | FALL |
|  | - | BFMVM162-12 | KC986041 | - | PB | FALL |
|  | - | BFMVM163-12 | KC986040 | - | PB | FALL |
|  | - | BFMVM164-12 | KC986039 | - | PB | FALL |
|  | - | BFMVM165-12 | KC986038 | - | PB | FALL |
|  | - | BFMVM166-12 | KC986062 | - | PB | FALL |
|  | - | BFMVM167-12 | KC986060 | - | PB | FALL |
|  | - | BFMVM168-12 | KC986059 | - | PB | FALL |
|  | - | BFMVM169-12 | KC986058 | - | PB | FALL |
|  | - | BFMVM170-12 | KC986057 | - | PB | FALL |
|  | - | BFMVM171-12 | KC985977 | - | PB | FALL |
|  | - | BFMVM172-12 | KC985976 | - | PB | FALL |
|  | - | BFMVM173-12 | KC985975 | - | PB | FALL |
|  | - | BFMVM174-12 | KC985974 | - | PB | FALL |
|  | - | BFMVM175-12 | KC985973 | - | PB | FALL |
|  | - | BFMVM176-12 | KC985972 | - | PB | FALL |
|  | - | BFMVM177-12 | KC985971 | - | PB | FALL |
|  | - | BFMVM178-12 | KC985970 | - | PB | FALL |
|  | - | BFMVM179-12 | KC985969 | - | PB | FALL |
|  | - | BFMVM180-12 | KC985967 | - | PB | FALL |
|  | - | BFMVM181-12 | KC985966 | - | PB | FALL |
|  | - | BFMVM182-12 | KC986056 | - | PB | FALL |
|  | - | BFMVM183-12 | KC985968 | - | PB | FALL |
|  | - | BFMVM184-12 | KC985986 | - | PB | FALL |
|  | - | BFMVM185-12 | KC985988 | - | PB | FALL |
|  | - | BFMVM186-12 | KC985990 | - | PB | FALL |
|  | - | BFMVM187-12 | KC985997 | - | PB | FALL |
|  | - | BFMVM188-12 | KC986001 | - | PB | FALL |
|  | - | BFMVM189-12 | KC986004 | - | PB | FALL |
|  | - | BFMVM190-12 | KC986006 | - | PB | FALL |
|  | - | BFMVM191-12 | KC986008 | - | PB | FALL |
|  | - | BFMVM192-12 | KC986015 | - | PB | FALL |
|  | - | BFMVM193-12 | KC986016 | - | PB | FALL |
|  | - | BFMVM194-12 | KC986019 | - | PB | FALL |
|  | - | BFMVM195-12 | KC986021 | - | PB | FALL |
|  | - | BFMVM286-12 | KC986061 | - | WV | SUMMER |
|  | - | BFMVM329-12 | KC986054 | - | WV | FALL |
|  | - | BFMVM330-12 | KC986053 | - | WV | FALL |
|  | - | BFMVM331-12 | KC986052 | - | WV | FALL |
|  | - | BFMVM332-12 | KC986051 | - | WV | FALL |
|  | - | BFMVM333-12 | KC986050 | - | WV | FALL |
|  | - | BFMVM334-12 | KC986035 | - | WV | FALL |
|  | - | BFMVM335-12 | KC986034 | - | WV | FALL |
|  | - | BFMVM336-12 | KC986033 | - | WV | FALL |
|  | - | BFMVM337-12 | KC986032 | - | WV | FALL |
|  | - | BFMVM338-12 | KC986031 | - | WV | FALL |
|  | - | BFMVM339-12 | KC986029 | - | WV | FALL |
|  | - | BFMVM340-12 | KC986028 | - | WV | FALL |
|  | - | BFMVM341-12 | KC986027 | - | WV | FALL |
|  | - | BFMVM342-12 | KC986025 | - | WV | FALL |
|  | - | BFMVM343-12 | KC986024 | - | WV | FALL |
|  | - | BFMVM344-12 | KC986017 | - | WV | FALL |
|  | - | BFMVM345-12 | KC986012 | - | WV | FALL |
|  | - | BFMVM346-12 | KC986000 | - | WV | FALL |
|  | - | BFMVM347-12 | KC985998 | - | WV | FALL |
|  | - | BFMVM348-12 | KC985991 | - | WV | FALL |
|  | - | BFMVM349-12 | KC985989 | - | WV | FALL |
|  | - | BFMVM350-12 | KC985985 | - | WV | FALL |
|  | - | BFMVM351-12 | KC985984 | - | WV | FALL |
|  | - | BFMVM352-12 | KC985983 | - | WV | FALL |
|  | - | BFMVM353-12 | KC985982 | - | WV | FALL |
|  | - | BFMVM354-12 | KC985981 | - | WV | FALL |
|  | - | BFMVM355-12 | KC985980 | - | WV | FALL |
|  | - | BFMVM356-12 | KC985979 | - | WV | FALL |
|  | - | BFMVM357-12 | KC985978 | - | WV | FALL |
| *Thalassiosira* sp. |  |  |  |  |  |  |
|  | - | BFMVM232-12 | KC986065 | - | WV | WINTER |
|  | - | BFMVM233-12 | KC986066 | - | WV | WINTER |
| *Thalassiosira* (tiny) sp. |  |  |  |  |  |  |
|  | - | BFMVM358-12 | KC985685 | - | WV | FALL |

^a^Isolates with no culture identifier are from the 2011 molecular-based survey.

^b^By visiting BOLD (www.boldsystems.org) and searching for identifiers, information regarding each isolate can be accessed, including: collection information (i.e., GPS coordinates, collector, etc.); confidence of the morphological identification of each sequence in the molecular-based survey; Culture Collection of Algae and Protozoa (CCAP) accession numbers if available; Ocean Genome Legacy (OGL) DNA bank accession numbers if available; primers used for amplification and sequencing; and trace files.

^c^PB = Passamaquoddy Bay and WV = The Wolves.

^d^Dates are indicated for colonies isolated for the culture-based DNA reference library (DRL) and season is indicated for isolates from the molecular-based survey. WINTER = January, April, May; SUMMER = June - September; and FALL = October, November, December.
